# Polymerase Chain Reaction Chips for Biomarker Discovery and Validation in Drug Development

**DOI:** 10.3390/mi16030243

**Published:** 2025-02-20

**Authors:** Dang-Khoa Vo, Kieu The Loan Trinh

**Affiliations:** 1 College of Pharmacy, Gachon University, 191 Hambakmoe-ro, Yeonsu-gu, Incheon 21936, Republic of Korea; kingsley688@gachon.ac.kr; 2 Bionano Applications Research Center, Gachon University, 1342 Seongnam-daero, Sujeong-gu, Seongnam-si 13120, Gyeonggi-do, Republic of Korea

**Keywords:** PCR chips, biomarker discovery, drug development, microfluidics, high-throughput screening, personalized medicine

## Abstract

Polymerase chain reaction (PCR) chips are advanced, microfluidic platforms that have revolutionized biomarker discovery and validation because of their high sensitivity, specificity, and throughput levels. These chips miniaturize traditional PCR processes for the speed and precision of nucleic acid biomarker detection relevant to advancing drug development. Biomarkers, which are useful in helping to explain disease mechanisms, patient stratification, and therapeutic monitoring, are hard to identify and validate due to the complexity of biological systems and the limitations of traditional techniques. The challenges to which PCR chips respond include high-throughput capabilities coupled with real-time quantitative analysis, enabling researchers to identify novel biomarkers with greater accuracy and reproducibility. More recent design improvements of PCR chips have further expanded their functionality to also include digital and multiplex PCR technologies. Digital PCR chips are ideal for quantifying rare biomarkers, which is essential in oncology and infectious disease research. In contrast, multiplex PCR chips enable simultaneous analysis of multiple targets, therefore simplifying biomarker validation. Furthermore, single-cell PCR chips have made it possible to detect biomarkers at unprecedented resolution, hence revealing heterogeneity within cell populations. PCR chips are transforming drug development, enabling target identification, patient stratification, and therapeutic efficacy assessment. They play a major role in the development of companion diagnostics and, therefore, pave the way for personalized medicine, ensuring that the right patient receives the right treatment. While this tremendously promising technology has exhibited many challenges regarding its scalability, integration with other omics technologies, and conformity with regulatory requirements, many still prevail. Future breakthroughs in chip manufacturing, the integration of artificial intelligence, and multi-omics applications will further expand PCR chip capabilities. PCR chips will not only be important for the acceleration of drug discovery and development but also in raising the bar in improving patient outcomes and, hence, global health care as these technologies continue to mature.

## 1. Introduction

Biomarkers reflect a wide array of biological processes, pathogenic conditions, and pharmacological responses and thus represent a cornerstone in both modern medicine and drug development [1,2,3]. Nowadays, these entities have proven useful as tools at different levels of the molecular hierarchy, including nucleic acids, proteins, metabolites, and other small molecules that help elucidate the mechanism of disease, stratify patient populations, predict treatment efficacy, and monitor therapeutic outcomes [4,5]. It has become increasingly clear to the pharmaceutical industry and academic research institutions over the past couple of decades that biomarkers can be instrumental in changing from traditional “one-size-fits-all” approaches to precision medicine paradigms [6,7]. Specifically, predictive biomarkers have been instrumental in the successes of targeted therapies, such as HER2 in breast cancer or PD-L1 in immuno-oncology, in facilitating the selection of patients most likely to benefit from specific treatments [8,9]. Biomarker discovery and validation are complex and resource-intensive processes, however, further complicated by inherent heterogeneity among diseases and inter-individual variability, along with the necessity for a robust method of validation that ensures reproducibility in all sorts of variable clinical settings [10,11,12]. The development of reliable biomarkers in drug development remains one of the critical but very challenging tasks that requires innovative technologies to surmount the existing hurdles [13,14]. More traditional approaches to biomarker discovery have included enzyme-linked immunosorbent assay (ELISA), Western blotting, and standard polymerase chain reaction (PCR) techniques, which have been in use in one form or another over several decades [15,16,17]. While effective in some situations, these traditional methods have some serious drawbacks that indeed limit their general applicability in today’s drug development pipelines. First, conventional methods are inherently laborious, requiring large sample volumes—a particular challenge when dealing with rare or otherwise difficult-to-obtain clinical specimens [18,19]. Second, these are not scalable in throughput that would be required to analyze such a large number of candidate biomarkers in parallel; hence, their utility in high-dimensional omics studies is also limited [20]. Third, traditional assays may possess suboptimal sensitivity and specificity, which at times makes the detection of low-abundance biomarkers and distinguishing closely related molecular species quite difficult [21]. Moreover, dynamic biological systems require technologies that enable real-time monitoring, something of which traditional methodologies are lagging [22,23]. This can imply longer biomarker validation times, higher costs, and a missed window of opportunity in a clinical setup for introducing therapeutic interventions. Such lacunae can only be mended through the integration of cutting-edge technologies that can blend speed, precision, and scalability together, allowing researchers to finally harvest the full potential of biomarker-driven drug development [24].

Owing to its extremely high sensitivity, specificity, and versatility, PCR technology has long been regarded as a gold standard for the detection and quantification of nucleic acids [25,26,27]. Recent advances in microfluidics have introduced PCR chips that are capable of miniaturizing and automating conventional PCR processes in chip-based format [28,29]. Using microfluidic designs, PCR chips provide parallel processing of several samples in nanoliter volumes, thus greatly increasing throughput while significantly reducing reagent consumption [30]. Such features make the PCR chip uniquely suited to address some limitations in conventional biomarker discovery methods [31,32]. In the context of biomarker discovery and validation, the following are the major advantages provided by a PCR chip. First, owing to the high sensitivity of PCR chips, they are capable of detecting low-abundance nucleic acid biomarkers, like rare mutations or circulating tumor DNA (ctDNA), which conventionally go undetected [33,34]. Second, the ability to multiplex many markers at a time contributes to an inclusive insight into intricate biological systems [35]. Third, the integration of real-time PCR capabilities enables dynamic monitoring of biomarker expression and thus provides insight into temporal changes that may be important for understanding disease progression or therapeutic responses [36,37]. In addition, PCR chips are highly amenable to emerging research needs, supporting applications such as single-cell analysis, digital PCR for absolute quantification, and integration with downstream analytical techniques [38,39]. All of these capabilities synergized to place PCR chips at the forefront as revolutionary tools in the speeding up of biomarker-driven drug development, bridging the gap between discovery and clinical application.

This review was focused on the overview of the role of PCR chips in biomarker discovery and validation, with special attention to their applications in drug development. By synthesizing recent developments in PCR chip technology, we want to bring to the fore their potential for solving some of the critical challenges associated with the identification, quantification, and validation of biomarkers. This review will also address current limitations and opportunities for innovation, providing a roadmap for future research in this fast-moving field. This review is very important not only to the academic community but also to stakeholders in the pharmaceutical and biotechnology industries, as well as to regulatory agencies and clinical researchers. This review strives to clarify the transformative potential of PCR chips for cross-disciplinary collaboration, which is going to be key in the adoption of those technologies in biomarker-driven drug development. Eventually, the integration of PCR chips into standard workflows holds the promise of accelerating the discovery of novel therapies, improving patient outcomes, and advancing the frontiers of precision medicine. We further elaborate on the principles of the technology behind the PCR chips, their application in biomarker discovery and validation, and analyze their impact on the drug development process. This review will balance the perspective of how these chips shape the future of healthcare and drug discovery by discussing opportunities as well as challenges with PCR chips.

## 2. Fundamentals of PCR Chips

### 2.1. What Are PCR Chips?

**Figure 1:**
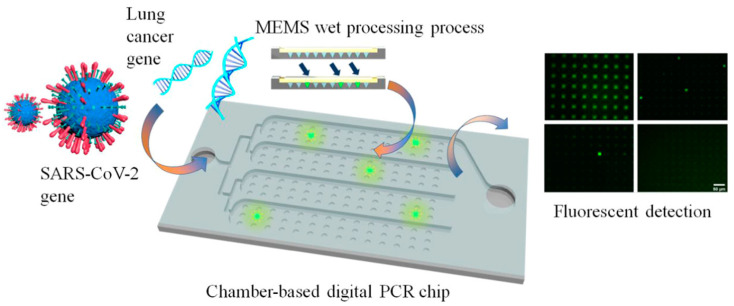
An example of a microchamber array digital PCR chip using silicon–glass material for SARS-CoV-2 virus and ultra-early-stage lung cancer marker quantitative detection. MEMS: microelectromechanical systems. Copyright ACS publisher (2012) [43].

PCR chips, or microfluidic PCR devices, are microdevices that execute the polymerase chain reaction process with exceptional efficiency and compactness [29,40,41]. These chips amalgamate microfluidics with molecular biology to facilitate accurate, rapid, and automated amplification of nucleic acids. They are particularly useful tools in biomarker discovery, diagnostics, and drug development. Microchannels and reaction chambers are normally etched or molded in substrates, including glass, silicon, and thermoplastics, or polymers such as polydimethylsiloxane (PDMS), poly(methyl methacrylate) (PMMA), polycarbonates (PC), or cyclic olefin copolymer (COC) (Figure 1) [42]. Figure 1 [43] illustrates an example of a microchamber array digital PCR chip fabricated using silicon–glass material. In their study, Sun et al. prepared a microarray cdPCR chip that enables high-throughput and high-sensitivity quantitative measurement of the SARS-CoV-2 virus gene and the mutant lung cancer gene, demonstrating its application in precise biomarker quantification for early diagnostics. These materials have become popular due to their excellent thermal conductivity, optical transparency, and resistance to most chemicals [44]. The conformation of chips minimizes the volume of reactions, usually in the nanoliter range, reducing consumption, and therefore the cost of the reagents, and increasing reaction kinetics [45]. The same principle of thermal cycling is used for designing PCR chips as that of conventional PCR [46]. Integrated microheaters and temperature sensors enable both fast and accurate thermal cycling; this is complemented by microfluidic channels that provide controlled reagent flow [47,48]. Most of them possess real-time fluorescence detectors, providing means for quantitative detection [49]. Some advantages these chips have over conventional PCR devices include reduced amplification time, reduced risk of contamination, being easily portable, miniaturization, and thus being scalable for high-throughput applications such as biomarker validation in drug development, point-of-care (POC) diagnostics, and environmental monitoring. The integration of microfluidics and PCR has facilitated rapid, accurate, and economical nucleic acid analysis.

### 2.2. Advantages of PCR Chips

PCR chips offer a host of advantages over traditional PCR methods, transforming nucleic acid amplification through their innovative design and functionality [50]. Probably the most important advantage is miniaturization. PCR chips work in reaction volumes within the nanoliter–picoliter range of volume, while the conventional PCR operates at microliter volumes [51,52]. This miniaturized format saves not only on reagents but also greatly facilitates compact and portable device-making. The latter feature makes them ideal for both POC testing and field applications [53]. Speed is another critical advantage, driven by the reduced thermal mass of the microfluidic design, enabling rapid heat transfer. Compared to conventional PCR, PCR chips are able to carry out the amplification cycles in fractions of time and often present results in minutes, not hours [54]. Moreover, the chips are more sensitive because they can regulate the reaction parameters precisely, including temperature and the mixing of reagents, thus minimizing sample loss and maximizing amplification efficiency [55]. These high-sensitivity chips are especially useful in the detection of low-abundance targets, such as rare genetic mutations or trace-level biomarkers that may be undetectable when using conventional methods [56]. Moreover, PCR chips save costs in terms of reagents and energy in addition to the saving of labor costs. Their multiplexing capability means that a number of reactions can be run on one chip at the same time, thus allowing further improvement of throughput and economy [57]. These abilities make them of special value for high-throughput applications, especially in biomarker screening, personalized medicine, and diagnostics [57]. Overall, PCR chips represent the revolutionary platform outperforming the traditional systems of PCR regarding speed, sensitivity, cost, and scalability.

### 2.3. Types of PCR Chips

**Figure 2:**
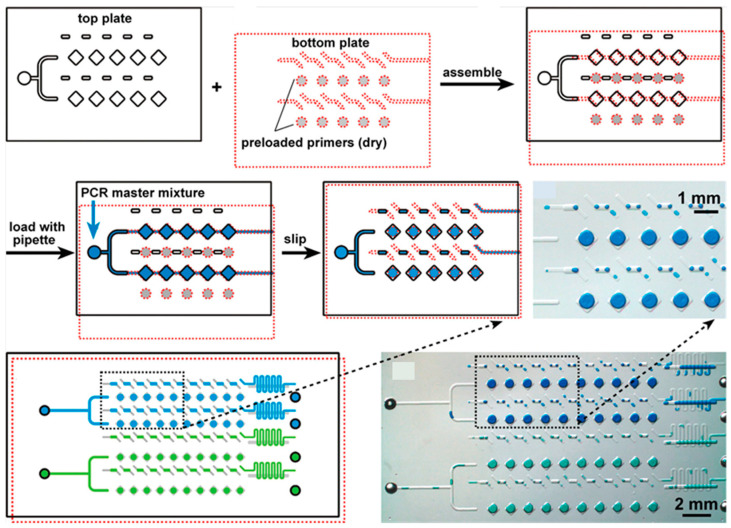
An overall schematic of assembly and operation of a nanoliter PCR SlipChip for *Staphylococcus aureus* detection using SlipChip. Copyright ACS (2010) [66].

There are different types of PCR chips designed to address the particular needs of molecular biology and diagnostics [58,59]. The most precise platforms are digital PCR (dPCR) chips, which partition the PCR reaction mixture into thousands of nanoliter-scale wells or droplets for absolute quantification of nucleic acids [60,61]. dPCR chips bring unparalleled sensitivity in the detection of low-abundance targets, such as rare mutations or viral DNA, by analyzing amplification in the presence or absence of a partition [62]. Otherwise, qPCR chips integrate fluorescence-based detection systems and monitor amplification in real time [63]. They are perfect for gene expression level quantification, especially for validating biomarkers and diagnosing infectious diseases quickly and accurately [64]. Multiplex PCR chips are designed for the amplification of multiple targets in one reaction and thus become a valuable tool when the throughput is high, as in the case of pathogen identification or genetic profiling [65,66] (Figure 2). Other special kinds of PCR chips include reverse-transcription PCR (RT-PCR) chips [67,68], which analyze RNA by converting it first to complementary DNA (cDNA), and isothermal PCR chips [69,70,71], where there is no thermal cycling since the methods of amplification at constant temperature are used. From clinical diagnostics and drug development to environmental monitoring and forensic analysis, different chip types are targeted for fulfilling various application needs, hence driving innovation in nucleic acid research.

## 3. The Role of PCR Chips in Biomarker Discovery

### 3.1. Identification of Novel Biomarkers

Well-known high-throughput screening (HTS) technologies make use of new biomarker identifications [72,73]. Comprehensive genetic and epigenetic markers have been used for identification and screening [74,75]. Thousands of genes, mutations, or epigenetic modifications are rapidly analyzed on a single platform coupled with state-of-the-art PCR chips [76,77], next-generation sequencing (NGS) [75,78,79], or microarray methodologies [80,81].

**Figure 3:**
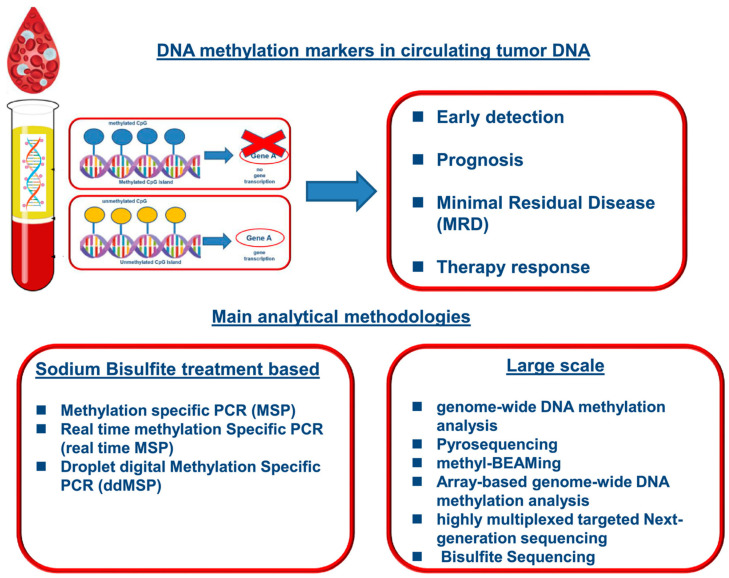
An overall illustration schematic of circulating tumor DNA methylation markers offers insights into early detection, prognosis, minimal residual disease, and therapeutic response. The primary analytical methodologies are founded either on PCR after sodium bisulfite (SB) conversion or on a comprehensive omic approach. For a clearer view of the figure details, please refer to the original source. Copyright Wiley (2021) [87].

Biomarkers are usually genetic markers like nucleotide polymorphisms (SNPs), copy number variations (CNVs), or gene fusions [82,83,84]. Diseases related to those genetic alterations are, among others, cancer, cardiovascular disorders, and infectious diseases. A few high-throughput chips can identify these biomarkers with a very high degree of sensitivity and specificity using only a few microliters of sample with, for example, high-throughput PCR chips [85,86]. Additionally, the epigenetic markers of DNA methylation patterns and histone modifications have recently been found to play very significant roles in both the pathogenesis of diseases and response to therapy [87] (Figure 3). HTS platforms, when coupled with bisulfite conversion techniques and quantitative PCR or sequencing, enable the precise mapping of methylation sites across the genome [88,89]. Of specific value, they represent an important avenue to be used in biomarker discovery for personalized medicine, since they provide disease-specific genetic and epigenetic alterations that could guide diagnosis, prognosis, and targeted therapies [90,91]. Moreover, high-throughput methodologies provide the capability to undertake large-scale investigations, such as genome-wide association studies (GWAS), aimed at the discovery of new biomarkers for genetically complex traits and conditions [92,93,94]. Altogether, HTS of genetic and epigenetic markers has become a keystone of modern biomedical research as a boost never previously attained, guaranteeing an unseen speed, accuracy, and depth of information.

### 3.2. Quantitative Analysis of Biomarkers

The field of biomarkers has become important for further diagnosis and prognosis, extending treatments for many diseases such as cancer, infectious diseases, and metabolic disorders [95,96]. Biomarker quantification is highly relevant to early detection, disease course monitoring, and personalized therapy design in cancer [97,98,99]. Most of the presently used determination tests for tumor markers, like ctDNA, microRNAs, and proteins such as carcinoembryonic antigen (CEA) and prostate-specific antigen (PSA), were developed using qPCR and techniques at the avant-garde, with liquid chromatography coupled to MS [100,101]. All of these methods provide a considerable level of sensitivity and specificity for cancer diagnosis at early stages and during follow-up after therapy [102]. Quantitative biomarker analysis underlies infectious diseases, providing rapid pathogen identification and assessment of disease severity [103]. For example, the quantification of viral RNA, as with HIV and SARS-CoV-2 infections during quantitative PCR (qPCR), is of great importance because it provides information on viral load in real time, guiding antiviral therapy and predicting outcomes [104]. Similarly, host inflammatory markers such as C-reactive protein (CRP) and interleukins may be used for assessing responses in bacterial or viral infection [105]. Biomarkers, such as glucose, insulin, lipids, and liver enzymes, have been quantitatively analyzed for the diagnosis and follow-up of metabolic disorders, including diabetes mellitus, dyslipidemia, and non-alcoholic fatty liver disease (NAFLD) [106]. Multiplex assays and point-of-care test advancements have greatly improved the efficiency and accessibility of biomarker quantification, thereby driving precision medicine across the board [107].

### 3.3. Single-Cell Analysis

Single-cell approaches are finding their place as a game-changing tool in the detection of rare biomarkers, with unprecedented insights into cellular heterogeneity and disease biology [108,109,110]. Other than bulk analysis, which averages signals across populations of cells, single-cell techniques enable the measurement of nucleic acids, proteins, and metabolites with precision at the level of a single cell [111,112]. In particular, this is important for identifying rare biomarkers, for example, from circulating tumor cells (CTCs), stem cells, or immune cells, which may mask their signals in heterogeneous cell populations [113]. For instance, single-cell analysis of cancer may find rare mutations, gene expression profiles, or epigenetic modifications that might drive tumor progression and metastasis or drug resistance [114].

Such technologies, including single-cell RNA sequencing (scRNA-seq) and droplet-based microfluidics, are able to provide a resolution that allows for the definition of subtle cell states or subpopulations, which are very important in diseases with complex cellular microenvironments, such as the tumor–immune interface or inflammatory conditions [115,116]. Single-cell analysis can be applied to understand cell infection due to a pathogen or characterize the immune response in infectious diseases at an intricate level that could help facilitate the development of vaccines and/or therapies [117]. For example, analytics on single cells can leverage rare biomarker detection and cellular diversity discovery for powerful applications in diagnostics, biomarker discovery, and therapeutic targeting based on precision medicine for various diseases [118].

## 4. The Role of PCR Chips in Biomarker Validation

### 4.1. Analytical Validation

One very important step in biomarker development is analytical validation, ensuring specificity, accuracy, and reproducibility characteristics that will be required for its clinical and research use [119]. The definition of specificity includes confirmation that the biomarker identifies the condition or biological state of interest and excludes all others, thus minimizing false positives [120]. This is of particular importance in diseases like cancer or autoimmune disorders, where overlapping molecular profiles may confound results. The biomarker of interest is exclusively detected and often verified using techniques like mass spectrometry, qPCR, or immunoassays [121,122]. Accuracy is defined as the closeness of the biomarker measure to its true value under standardized conditions using well-characterized reference materials, calibration standards, and method comparisons [123]. For example, in pharmacokinetics, high accuracy for the quantification of small-molecule biomarkers has been achieved using liquid chromatography-tandem mass spectrometry LC-MS/MS [124]. Biomarker measurements are expected to be highly reproducible in different laboratories, instruments, and experimental conditions, that is, with exhaustive testing across many batches, operators, and environments for establishing their robustness [125]. Automation in microfluidics and multiplex platforms reduces the variability introduced by manual operations, thus enhancing reproducibility [126]. These various validation processes create a confidence factor in biomarker performance and a sense of their reliability in disease diagnosis, prognosis, therapeutic monitoring, and regulatory approval [127]. Analytical validation bridges the gap between biomarker discovery and clinical application.

### 4.2. Clinical Validation

**Figure 4:**
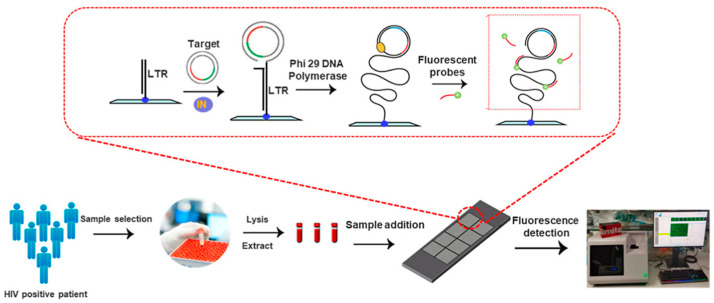
Schematic diagram of the HIV integrase-specific DNA biosensing platform based on the rolling circle amplification (RCA) technique for multiple amplifications for HIV detection. LTR: long terminal repeat sequences. Copyright ACS (2024) [140].

Clinical validation is one very important step in translating biomarkers into clinical practice [128]. It is essential in ensuring the reliability and relevance of biomarkers across diverse patient populations. Because of their high sensitivity, specificity, and scalability, PCR chips have become imperative in this process, thus enabling the robust validation of biomarkers in large-scale studies. These chips can analyze several biomarkers contemporaneously and, thus, are the consummate chips for broad-based validation efforts in complex diseases such as cancer, infectious diseases, and genetic disorders [129]. In cancer, for example, PCR chips now allow for the validation of ctDNA, gene mutations, or expression profiles such as BRCA1, TP53, or KRAS mutations through many thousands of patient samples for their potential for early detection, prognostication, and therapy selection [130,131,132,133,134]. For example, for the validation of viral and bacterial markers relevant to infectious diseases such as SARS-CoV-2 RNA or HIV viral load across diverse populations, it is necessary for diagnostic accuracy and clinical utility to be demonstrated [135,136]. On the other side, for the clinical validation of PCR chips, there are high-throughput capabilities, a low sample requirement and reagent consumption, real-time data, and integrated fluorescence-based detection [137,138]. This will not only ensure faster validation but will also enhance data reliability and reproducibility. Because the PCR chips accelerate large-scale validation in patient populations, they fast-track the integration of novel biomarkers into routine diagnostics and personalized treatment strategies [139]. For instance, Figure 4 [140] illustrates a DNA biosensor platform constructed to be specific and sensitive for the detection of HIV. The sensor combines a rolling circle amplification (RCA)-based sensor and a portable fluorescent detector, using the HIV integrase enzyme activity as a marker for amplification of the signal cascade with a detection range of 0.125 CFU/μL. The platform was highly sensitive in HIV detection from clinical samples correlated with CD4+ lymphocyte counts and had the potential for rapid screening, diagnosis, monitoring of therapy, and implementation of efficient HIV prevention programs.

PCR chips have emerged as valuable instruments in clinical research for facilitating the detection of biomarkers, for example, circulating DNA in cancer patients. In one such case, a study reports the utility of a chip-based dPCR platform to identify circulating DNA in 34 metastatic colorectal cancer patients [141]. The platform demonstrated 69% detectability of ctDNA and revealed high correlations of cfDNA with ctDNA levels. Elevated levels of cfDNA and detectable levels of ctDNA were associated with the poorest overall survival. The results illustrate the potential that chip-based dPCR possesses as an informative, non-invasive method of prognostication in the management of metastatic colorectal cancer.

## 5. Applications of PCR Chips in Drug Development

### 5.1. Target Identification and Validation

**Figure 5:**
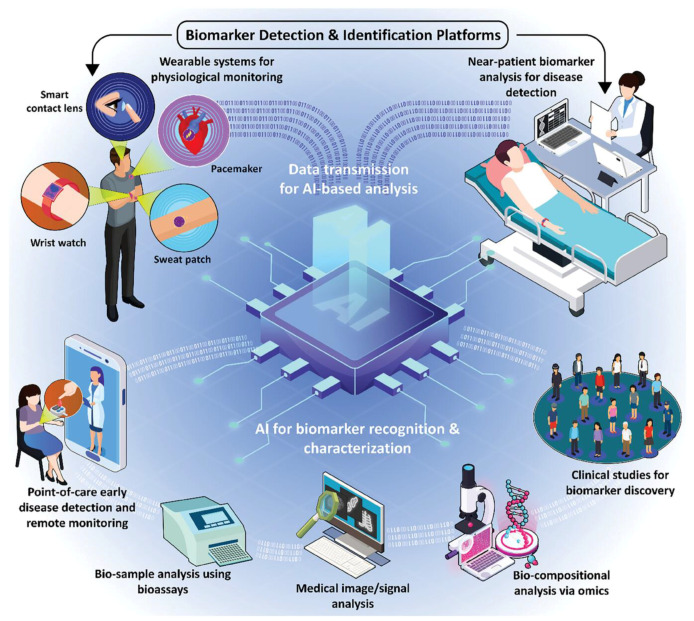
An overall schematic representation of biomarker detection and identification platforms by combining point-of-care microdevice systems and intelligent technologies. Copyright Wiley (2024) [145].

Target identification and its validation are some of the important steps in drug development wherein biomarkers identify druggable targets that can be modulated for therapeutic benefit [142,143]. Biomarkers can be specific proteins, gene mutations, or molecular pathways that can give insight into the identification of potential targets involved in disease progression [144,145] (Figure 5). Biomarkers can indicate alterations in signaling pathways, for example, changes that are critical for cancerous cell growth, as in the case of EGFR or PI3K activity, representing a druggable target activity necessary for tumor growth and survival [146,147]. Biomarkers that indicate some form of alterations in lipid metabolism or insulin resistance are biomarkers that characterize novel drug interventions against metabolic disorders like diabetes [148]. Biomarker validation confirms that a target is implicated not only in the mechanism of the disease but is also modifiable by therapeutic agents once potential druggable targets are identified [149,150]. Some of these biomarkers have been validated with techniques such as RNA sequencing, mass spectrometry, and immunohistochemistry for their presence in patient samples and functional relevance [151,152]. Biomarkers can also be used to assess drug candidates based on their capabilities to change a target to provide treatment efficacy insights [153]. Biomarker-validated targets also enhance the high-throughput screening of compounds and speed up drug development [154]. Therefore, small molecules, biologics, and gene therapies with precise modulation of these targets could be identified. Biomarkers play a very crucial role in both target identification and target validation and form the pathway to the creation of targeted therapies [155].

### 5.2. Patient Stratification

The rationale behind personalized medicine depends on timely, more effective options of treatment through the biomarker-driven stratification of patients [156,157]. Biomarkers in this regard are genetic, proteomic, or epigenetic entities indicative of subtypes of a disease and/or of disease progression, with expected responses to therapy included, classifying patients according to molecular profiling [158]. An example would be in cancer, where genetic mutations or expression patterns such as EGFR or HER2 are involved in facilitating the identification of patients most likely to benefit from targeted therapies or immunotherapies [159,160]. In metabolic disorders, biomarkers of insulin sensitivity or lipid metabolism can classify patients according to specific disease mechanisms for guiding the choice of interventions, for example, antidiabetic drugs or lifestyle modifications [161]. Biomarker-based patient stratification not only enhances treatment efficacy but also decreases the risk of drug-related side effects, limiting them to narrow circles of patients who benefit from a certain therapy [162]. In oncology, it could be envisioned that patients bearing specific genetic alterations will be treated with better outcomes by using targeted therapies [163,164]. Biomarker-based stratification also allows for the real-time monitoring of the responses to particular treatments and, consequently, tuning therapies [165]. This is a precision medicine approach powered by biomarkers, making sure patients receive the most appropriate treatment and ensuring outcomes with minimal unnecessary interventions.

### 5.3. Monitoring Drug Response

Real-time monitoring of drug response is among one of the most paramount modalities that could improve therapeutic efficacy while minimizing toxicity in patients [166]. Biomarkers help in providing real-time data about how a patient is responding to various treatments [167]. For instance, the measurement of ctDNA or certain protein biomarkers will yield direct feedback, for example, whether tumor shrinkage or the development of resistance is taking place during a course of cancer treatment, thus allowing changes in treatment to be made by the clinician [168,169]. Real-time detection using PCR-based methods of viral load provides information, in infectious diseases, on the efficacy of anti-viral drugs, therefore allowing for the needed dose or therapeutic strategy modifications [170,171]. Biomarkers help quantify the toxic nature of the effects brought about by treatment [172]. Monitoring certain biomarkers in liver or kidney function, such as alanine aminotransferase (ALT) or levels of creatinine, can serve as early warnings for clinicians concerning adverse reactions so that severe complications can be avoided [173]. These biomarkers have wide applications in cases of chemotherapy and immunotherapy because elevated cytokine levels or immune cell profiles signal immune-related adverse events and allow for timely interventions [174]. Monitoring real-time drug response provides not only the best possible therapy for the patients but also alerts doctors early in the course of therapy regarding any side effects so that doses can be altered or an alternative therapy administered [175]. It can greatly improve the outcomes for the patient, decrease the incidence of adverse reactions, and improve the general efficiency of drug development.

### 5.4. Companion Diagnostics

The main purpose of companion diagnostics within the development process of a diagnostic tool along with targeted therapy is to ensure proper therapy to the proper patient at the right time [176,177]. Companion diagnostic evaluations in most cases involve biomarkers, and they serve to determine those patients who would benefit most from a specific form of treatment, for example, oncology, rare disease, or chronic diseases [178,179]. In this context, companion diagnostics are applied for the detection of genetic mutations of EGFR or BRAF genes, acting as biomarkers, which allow for the application of specific treatments, while drugs will be of real value in treating only the selected patients who host such genetic alterations [180]. Therefore, companion diagnostics could guarantee selection for treatment of those in whom maximal therapeutic effects could be accrued and at the same time diminish harm by not selecting patients unnecessarily due to potential adverse drug effects. Because companion diagnostics are being developed in a coordinated manner with the targeted therapies, medicine could be more personalized and the effectiveness of treatments increased while minimizing risks associated with inefficient therapies [181]. Companion diagnostics that have been co-developed have been acknowledged by regulatory agencies like the FDA, and drugs together with their companion diagnostics are approved for use in order to ensure proper stratification of patients [182]. Such a holistic approach offers better patient outcomes and shortens drug development times, as therapies can be monitored in real time for effectiveness and support the paradigm of precision medicine.

## 6. Current Challenges and Limitations

### 6.1. Technological Barriers

Despite these tremendous advances in biomarker-driven drug development and personalized medicine, several technological barriers are still in place that limit the full realization of such approaches. First, there is integration across the other omics technologies, a suite of genomics, proteomics, transcriptomics, and metabolomics, which all together generate huge amounts of data with intensive complexity to be integrated holistically and properly interpreted [183,184]. The lack of standardized combinatory platforms of omics data hampers the full unmasking molecular complexity of diseases, and this gap requires further bioinformatics tool development able to integrate multi-omics data for the more accurate identification of biomarkers and better stratification of patients [185,186]. Scalability is another challenge of significant importance. Whereas the high throughput and sensitivity afforded by PCR chips, microarrays, and NGS come with great cost, scaling these technologies for large clinical cohorts or high-volume screening is exceedingly cumbersome [187]. When using these technologies, efforts have to be made to ensure that there is reproducibility of results across diverse patient populations with minimal technical variability if their use in widespread clinical adoption is considered. Finally, cost is a significant barrier to the widespread use of advanced biomarker technologies [188]. Combining more than one type of omics technology with a high-throughput platform and state-of-the-art bioinformatics support is very expensive [189]. Thus, this limits access to such tools in low-resource settings, which makes it challenging to achieve full-scale implementation of precision medicine strategies in many parts of the world. Overcoming these barriers will depend on sustained technological innovation, standardization, and cost-reduction strategies to improve the accessibility and scalability of such approaches.

Closed-loop microfluidic devices are an important improvement to overcome PCR chip technology challenges such as contamination, reproducibility, and clinical workflow integration. Closed-loop systems take advantage of sealed environments to reduce contamination during PCR amplification and on-chip internal controls and automation to enhance reproducibility across experiments. For instance, Kim et al. [190] created an advanced in vitro platform to study dopamine (DA) homeostasis and its role in Parkinson’s Disease (PD) progression. The platform includes a microfluidic device to culture DAergic neurons, an optical detection apparatus for DA sensing, and a closed-loop automatic control system to deliver medication. Through efficient control of DA homeostasis through SH-SY5Y neuroblastoma cells, the platform facilitates real-time monitoring, drug screening, and PD research. This underscores the potential of microfluidic innovations to address core challenges and reshape healthcare applications.

### 6.2. Biological Challenges

Probably the most relevant biological challenges are biomarker heterogeneity and variability, each of which significantly influences biomarker discovery, validation, and clinical application [191,192]. Biomarker heterogeneity reflects the heterogeneity in biomarker expression in a population of cells, tissues, or individuals [193]. The origin of such variability may be genetic, epigenetic, or environmental [194]. Sometimes, it also varies in different stages or subtypes of disease or even in different parts of one tumor. In cancer, for example, the expression of any one biomarker, such as HER2 or EGFR, can easily span a broad range between different tumor subtypes, or even within metastatic sites, making searching for broadly applicable biomarkers for diagnosis or treatment challenging [195]. This heterogeneity may well promote false negatives or positives, limiting the reliability and specificity of biomarkers in the clinical setting [196]. In addition, biomarker variability is another challenge. The biomarkers may temporally vary as a result of changes in disease progression, therapeutic intervention, or individual patient characteristics [197]. Such temporal variability further complicates the establishment of clear thresholds for biomarker levels, especially in drug response or recurrence monitoring. Moreover, such inter-individual variability may relate to genetic differences in biomarker expression, which would predispose patients to different responses to treatment and therefore affect the precision of personalized medicine [198]. Overcoming these challenges requires the development of more refined methods of biomarker analysis, including advanced technologies that take into consideration intra- and inter-patient variability for correct and reliable clinical application.

### 6.3. Regulatory and Ethical Considerations in Biomarker-Based Drug Development

Regulatory aspects are of great importance in the validation of biomarkers, and most importantly, in clinical use, whereby regulatory agencies such as the U.S. Food and Drug Administration (FDA) and the European Medicines Agency (EMA) have stringent guidelines for biomarker validation [199,200]. Adherence to these guidelines ensures that not only are biomarkers scientifically sound but also that they are safe and effective for patients. Biomarkers have to be analytically validated according to the FDA and EMA for specificity, accuracy, and reproducibility in clinical use [201]. Another way of establishing uniformity across laboratories and within clinical trials themselves is through clear articulation of protocols about biomarker testing, sample collection, and data analysis. Major demands from both agencies relate to clinical validation requirements under which it can be tested in large patient populations for its potential clinical utility, such as diagnostics, disease progression prediction, or therapeutic monitoring [202]. Biomarker-based diagnosis and treatment, on the other hand, rely on “Good Laboratory Practice and Good Manufacturing Practice” to ensure that the practices of laboratory procedures and manufacturing processes are quality controlled [203,204]. Any regulatory submission must be documented in full, including all steps of validation and performance characteristics, including clinical trial data. Similarly, the validation of PCR-based assays or any other technologies for biomarker quantification should also be performed. Biomarkers that have been validated in conformity with FDA/EMA guidelines will ensure their safe entry into clinical practice for diagnosis and treatment [205].

Biomarker-based drug development is critical with regard to regulatory and ethical considerations in ensuring that biomarker identification, validation, and application are responsibly carried out regarding safety concerns [203]. Thus, the FDA and EMA have adopted an approach that requires broad data, providing evidence of the safety, efficacy, and clinical utility of biomarkers before approval for use either in drug development or diagnostics [206]. This would mean rigorous testing through analytical validation, to establish the biomarkers and indeed measure what they are supposed to, and clinical validation to establish the predictive relevance of the biomarkers to disease outcome or treatment response. Companion diagnostics are biomarkers utilized in guiding treatment decisions and generally need regulatory approval of the diagnostic test and its use therapy to ensure the safety and efficacy of the combination in patients [207]. There are ethical considerations in using biomarkers, including the question of patient privacy and informed consent, especially when genetic information is at stake [208,209]. Patients have a right to full information on the use and storage of and access to their biomarker data [210]. In addition, personalized medicine should address how biomarkers and associated health disparities might be applied to make the therapies available for all different population groups without consideration of their socio-economic status or their geographical location [211]. For instance, there should be ethical guidelines that regulate the use of information obtained from biomarkers in a manner likely to discriminate or lead to stigmatization [212,213,214]. Addressing these regulatory and ethical challenges is essential for advancing biomarker-based drug development while safeguarding public trust.

## 7. Emerging Trends and Future Perspectives

### 7.1. Integration with Artificial Intelligence and Machine Learning

**Figure 6:**
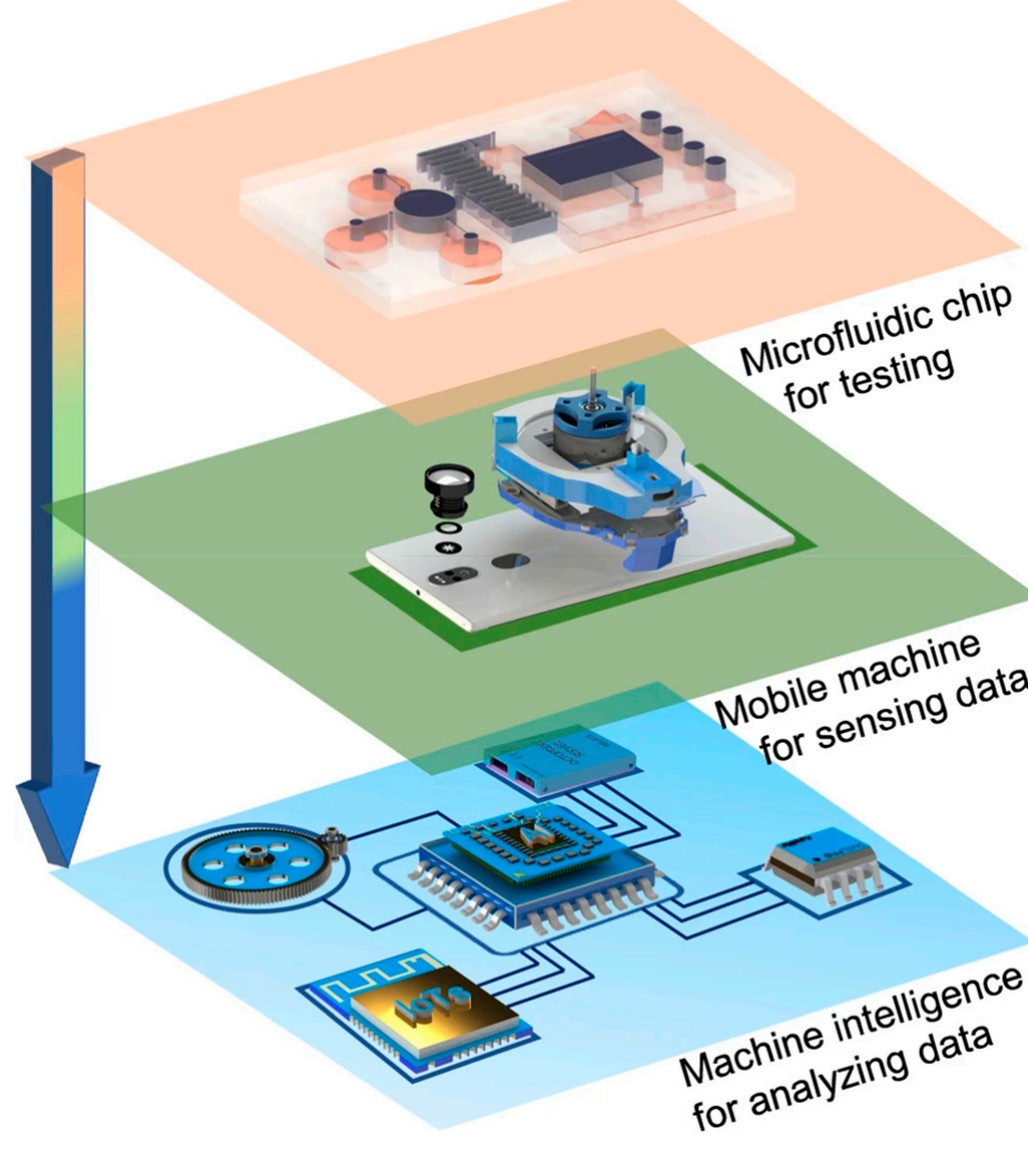
An example of smartphone-based platforms integrating microfluidic detection with image-based artificial intelligence in point-of-care testing applications. Copyright *Nature* (2023) [216].

Biomarker discovery can be integrated with artificial intelligence (AI) and machine learning (ML), making for a complete reevaluation of how one approaches biomarker identification, validation, and clinical use [215,216] (Figure 6). AI/ML algorithms can analyze the immense quantity of complicated and high-dimensional data that are the output of investigations into genomics, proteomics, metabolomics, and imaging in order to reveal patterns and associations not feasible or too cumbersome to determine using conventional approaches [217]. By applying unsupervised learning, for instance, AI models are able to define new biomarkers of disease onset, progression, and treatment response without a priori hypotheses, thus accelerating the process of biomarker discovery [218,219]. For instance, AI-driven methods have been applied in predictive biomarker identification for cancer immunotherapy based on gene expression analysis with a view to more appropriate patient stratification for clinical trials [220]. AI and ML further make sure that biomarker panel validation and optimization are much stronger in terms of the power of discriminability between disease states, thus minimizing false positives and false negatives [221]. Deep learning, a form of AI, holds a specific appeal for uncovering complicated features from medical images such as computed tomography scans or histopathology slides to identify biomarkers associated with early disease or therapeutic response [222]. Furthermore, AI may allow for the integration of multi-omics data, thus enabling a more holistic understanding of disease mechanisms [223]. Eventually, the integration of AI/ML into biomarker discovery pipelines accelerates biomarker identification, enhances diagnostic precision, and propels personalized medicine through deeper insights into disease biology and therapeutic responses.

### 7.2. Multi-Omics Approaches

Multi-omics, which involves a combination of genomics, transcriptomics, and proteomics, has revolutionized the understanding of complex diseases by promoting biomarker discovery. Such multi-omics integrates information from various layers of biological processes, hence affording a holistic, more nuanced view of the mechanisms responsible for the disease process [224]. Whereas genomics will indicate genetic mutations, variations, and epigenetic changes driving diseases, transcriptomics will disclose gene expression patterns reflecting cellular responses either to disease or treatment [225]. Proteomics extends this by identifying and quantifying the proteins that carry out cellular functions, providing direct evidence of disease processes at the functional level [226]. PCR chip technology is important in enabling the integration of multi-omics data through the provision of high-throughput, sensitive, and cost-effective platforms for genomics and transcriptomics [227]. PCR chips are also extremely useful for studying complex biological samples, providing an opportunity to amplify and quantify, simultaneously, various genetic markers and gene expression profiles [228,229]. Coupled with other proteomics technologies, such as mass spectrometry or antibody-based assays, this provides an enabling workflow from genetic and transcriptomic information to functional protein expression [230,231]. Thus, integration might highlight novel biomarkers for disease diagnosis, prognosis, and treatment response while being useful in the stratification of patients to personalized medicine with better precision. The synergistic interaction of multi-omics technologies with PCR chips has advanced biomarker discoveries and thereby accelerated targeted therapy development.

### 7.3. Advancements in Chip Design

Improvements in chip design, especially 3D-printed chips, lab-on-a-chip devices, and portable PCR systems, have great potential for biomarker analysis and diagnostics because they can improve accessibility, efficiency, and scalability significantly [232,233]. 3D printing in chip design introduces new frontiers with the development of customized chips at low cost and possessing complex microfluidic networks that can handle diverse biological samples [234,235]. In a rather short time, these chips can be mass-manufactured, enabling several different analytical functionalities such as DNA amplification, separation, and detection on one chip [236]. Thus, lab-on-a-chip devices are particularly suitable for point-of-care diagnostics, especially in settings with limited resources [237]. Lab-on-a-chip devices integrate multiple functionalities traditionally carried out separately within a conventional laboratory onto a compact, single platform, allowing high throughput with minute volumes of both reagent and sample consumption [238,239]. These devices combine fluid handling, biomarker detection, and sometimes even data analysis in one package, thus enabling real-time results with high sensitivity and accuracy [240]. A wide exploration of their applications is being carried out in personalized medicine, infectious disease diagnostics, and environmental monitoring [241]. Another major development is portable PCR systems that permit faster genetic analysis at the site of requirement. These systems embody PCR amplification and detection with often-combined sample preparation in a compact, user-friendly format [242]. This portability is very useful for field diagnostics and monitoring infectious diseases or genetic conditions in real time without any need for centralized laboratory infrastructure [243]. Combined, these advances in chip design have transformed diagnostics, improving the speed, precision, and accessibility of biomarker-based analysis both in clinical and field settings.

### 7.4. Applications Beyond Drug Development

Biomarker-driven technologies, like PCR chips or other diagnostic platforms, represent much more than plain drug development and instead touch the core of precision medicine and global health [243]. Biomarkers in precision medicine provide possibilities for tailoring the treatment approaches of a particular patient in light of genetic, proteomic, and environmental profiling to enable choosing those therapies likely to be more effective with reduced adverse effects and thus achieve an improvement in patient outcomes [244]. Biomarkers have a critical role in determining the choice of treatment for cancer patients, depending on genetic mutations, intending to utilize targeted therapies addressing those particular mutations [245]. In metabolic diseases like diabetes, biomarkers help in the subclassification of patients according to subtypes of disease and direct personalized treatment [246]. Biomarker-based diagnostics, especially, serve infectious diseases for global health initiatives dealing with tuberculosis, malaria, and HIV amongst the resource-poor [247]. Portable polymerase chain reaction systems and lab-on-a-chip devices present a suitable cost-effective means of early disease diagnosis, real-time monitoring, and treatment via timely interventions while offering better management of the disease [248]. These technologies are crucial to epidemiological surveillance in offering rapid and extensive population screening, which enables observation of the spread of outbreaks of disease and advice on policies relating to public health. Biomarker technologies will continue to develop new diagnostic capabilities and personalized approaches that drive innovation in patient care and improvements in global health.

## 8. Conclusions

PCR chips have become key instruments of biomarker discovery and validation by combining unprecedented sensitivity with scalability and cost efficiency. Such technologies enable high-throughput, precise analyses of biomarkers at the level of the genome, transcriptome, and proteome. They therefore provide the most valuable information concerning disease-related molecular processes and therapeutic responses. Integration with emerging technologies, lab-on-a-chip devices, and portable PCR systems now enables biomarker testing by on-site researchers and clinicians, therefore saving time for real-time diagnoses and improving access in both clinical and field settings. This is particularly a transformative capability for precision medicine, where one’s molecular profile may be leveraged to realize personalized therapeutic regimens, optimizing therapeutic efficacy with the fewest adverse effects. However, the capabilities of PCR chips go well beyond research into crucial ways of accelerating drug development by simplifying the biomarker validation process. The ability of PCR chips to identify and validate biomarkers for a wide array of diseases, including cancer, infectious diseases, and metabolic disorders, allows for the efficient development of targeted therapies together with companion diagnostics. This in turn will ensure better patient outcomes because treatments can then be prescribed, tailored to the particular characteristics of one specific patient’s disease. Regarding technology and biological variability, not to mention regulatory issues, the greatest challenges to the commercial realization of PCR chips have yet to be resolved. Urgent transdisciplinary research and innovation in collaboration should be welcomed to help overcome such obstacles. This will include even more advanced, scalable, and accessible solutions from academia, industry, and regulatory bodies that enhance the role of PCR chips in biomarker discovery, drug development, and personalized healthcare, thus changing patient care on a global level.

## References

[1] Mayeux R. (2004). Biomarkers: Potential uses and limitations. NeuroRx.

[2] Chikkaveeraiah B.V., Bhirde A.A., Morgan N.Y., Eden H.S., Chen X. (2012). Electrochemical Immunosensors for Detection of Cancer Protein Biomarkers. ACS Nano.

[3] Haes A.J., Chang L., Klein W.L., Van Duyne R.P. (2005). Detection of a Biomarker for Alzheimer’s Disease from Synthetic and Clinical Samples Using a Nanoscale Optical Biosensor. J. Am. Chem. Soc..

[4] Frank R., Hargreaves R. (2003). Clinical biomarkers in drug discovery and development. Nat. Rev. Drug Discov..

[5] Hanash S.M., Baik C.S., Kallioniemi O. (2011). Emerging molecular biomarkers—Blood-based strategies to detect and monitor cancer. Nat. Rev. Clin. Oncol..

[6] Hartl D., de Luca V., Kostikova A., Laramie J., Kennedy S., Ferrero E., Siegel R., Fink M., Ahmed S., Millholland J. (2021). Translational precision medicine: An industry perspective. J. Transl. Med..

[7] Seyhan A.A., Carini C. (2019). Are innovation and new technologies in precision medicine paving a new era in patients centric care?. J. Transl. Med..

[8] Derouane F., van Marcke C., Berlière M., Gerday A., Fellah L., Leconte I., Van Bockstal M.R., Galant C., Corbet C., Duhoux F.P. (2022). Predictive biomarkers of response to neoadjuvant chemotherapy in breast cancer: Current and future perspectives for precision medicine. Cancers.

[9] Gohil S.H., Iorgulescu J.B., Braun D.A., Keskin D.B., Livak K.J. (2021). Applying high-dimensional single-cell technologies to the analysis of cancer immunotherapy. Nat. Rev. Clin. Oncol..

[10] Goossens N., Nakagawa S., Sun X., Hoshida Y. (2015). Cancer biomarker discovery and validation. Transl. Cancer Res..

[11] Ilyin S.E., Belkowski S.M., Plata-Salamán C.R. (2004). Biomarker discovery and validation: Technologies and integrative approaches. Trends Biotechnol..

[12] McDermott J.E., Wang J., Mitchell H., Webb-Robertson B.-J., Hafen R., Ramey J., Rodland K.D. (2013). Challenges in biomarker discovery: Combining expert insights with statistical analysis of complex omics data. Expert Opin. Med. Diagn..

[13] Dieterle F., Marrer E. (2008). New technologies around biomarkers and their interplay with drug development. Anal. Bioanal. Chem..

[14] Gupta S., Venkatesh A., Ray S., Srivastava S. (2014). Challenges and prospects for biomarker research: A current perspective from the developing world. Biochim. Biophys. Acta (BBA)-Proteins Proteom..

[15] Solier C., Langen H. (2014). Antibody-based proteomics and biomarker research—Current status and limitations. Proteomics.

[16] Rifai N., Gillette M.A., Carr S.A. (2006). Protein biomarker discovery and validation: The long and uncertain path to clinical utility. Nat. Biotechnol..

[17] Peng P., Liu C., Li Z., Xue Z., Mao P., Hu J., Xu F., Yao C., You M. (2022). Emerging ELISA derived technologies for in vitro diagnostics. TrAC Trends Anal. Chem..

[18] Jung B., Adeli K. (2009). Clinical laboratory reference intervals in pediatrics: The CALIPER initiative. Clin. Biochem..

[19] Crowther L.M., Poms M., Plecko B. (2018). Multiomics tools for the diagnosis and treatment of rare neurological disease. J. Inherit. Metab. Dis..

[20] Misra B.B., Langefeld C., Olivier M., Cox L.A. (2019). Integrated omics: Tools, advances and future approaches. J. Mol. Endocrinol..

[21] Filkins L.M., Bryson A.L., Miller S.A., Mitchell S.L. (2020). Navigating clinical utilization of direct-from-specimen metagenomic pathogen detection: Clinical applications, limitations, and testing recommendations. Clin. Chem..

[22] Vojinović V., Cabral J.M.S., Fonseca L.P. (2006). Real-time bioprocess monitoring: Part I: In situ sensors. Sens. Actuators B Chem..

[23] Teymourian H., Tehrani F., Longardner K., Mahato K., Podhajny T., Moon J.-M., Kotagiri Y.G., Sempionatto J.R., Litvan I., Wang J. (2022). Closing the loop for patients with Parkinson disease: Where are we?. Nat. Rev. Neurol..

[24] Tan D.S.W., Thomas G.V., Garrett M.D., Banerji U., De Bono J.S., Kaye S.B., Workman P. (2009). Biomarker-driven early clinical trials in oncology: A paradigm shift in drug development. Cancer J..

[25] Oliveira B.B., Veigas B., Baptista P.V. (2021). Isothermal amplification of nucleic acids: The race for the next “gold standard”. Front. Sens..

[26] Zhu H., Zhang H., Xu Y., Laššáková S., Korabečná M., Neužil P. (2020). PCR past, present and future. Biotechniques.

[27] Navarro E., Serrano-Heras G., Castaño M.J., Solera J. (2015). Real-time PCR detection chemistry. Clin. Chim. Acta.

[28] Zhang C., Xu J., Ma W., Zheng W. (2006). PCR microfluidic devices for DNA amplification. Biotechnol. Adv..

[29] Zhang C., Xing D., Li Y. (2007). Micropumps, microvalves, and micromixers within PCR microfluidic chips: Advances and trends. Biotechnol. Adv..

[30] Park S., Zhang Y., Lin S., Wang T.-H., Yang S. (2011). Advances in microfluidic PCR for point-of-care infectious disease diagnostics. Biotechnol. Adv..

[31] Mohammed M.-I., Desmulliez M.P.Y. (2011). Lab-on-a-chip based immunosensor principles and technologies for the detection of cardiac biomarkers: A review. Lab Chip.

[32] Wu L., Qu X. (2015). Cancer biomarker detection: Recent achievements and challenges. Chem. Soc. Rev..

[33] Diao Z., Han Y., Zhang R., Li J. (2020). Circulating tumour DNA: A new biomarker to monitor resistance in NSCLC patients treated with EGFR-TKIs. Biochim. Biophys. Acta (BBA) Rev. Cancer.

[34] Bronkhorst A.J., Holdenrieder S. (2023). The changing face of circulating tumor DNA (ctDNA) profiling: Factors that shape the landscape of methodologies, technologies, and commercialization. Med. Genet..

[35] Behura S.K. (2006). Molecular marker systems in insects: Current trends and future avenues. Mol. Ecol..

[36] Bamodu O.A., Chung C.-C., Pisanic T.R. (2023). Harnessing liquid biopsies: Exosomes and ctDNA as minimally invasive biomarkers for precision cancer medicine. J. Liq. Biopsy.

[37] Van de Sande B., Lee J.S., Mutasa-Gottgens E., Naughton B., Bacon W., Manning J., Wang Y., Pollard J., Mendez M., Hill J. (2023). Applications of single-cell RNA sequencing in drug discovery and development. Nat. Rev. Drug Discov..

[38] Brennan D., Justice J., Corbett B., McCarthy T., Galvin P. (2009). Emerging optofluidic technologies for point-of-care genetic analysis systems: A review. Anal. Bioanal. Chem..

[39] Yin J., Suo Y., Zou Z., Sun J., Zhang S., Wang B., Xu Y., Darland D., Zhao J.X., Mu Y. (2019). Integrated microfluidic systems with sample preparation and nucleic acid amplification. Lab Chip.

[40] Zhang C., Xing D. (2007). Miniaturized PCR chips for nucleic acid amplification and analysis: Latest advances and future trends. Nucleic Acids Res..

[41] Wang J., Chen Z., Corstjens P.L.A.M., Mauk M.G., Bau H.H. (2006). A disposable microfluidic cassette for DNA amplification and detection. Lab Chip.

[42] Cortese B., Mowlem M.C., Morgan H. (2011). Characterisation of an irreversible bonding process for COC–COC and COC–PDMS–COC sandwich structures and application to microvalves. Sens. Actuators B Chem..

[43] Sun Y., Huang Y., Qi T., Jin Q., Jia C., Zhao J., Feng S., Liang L. (2022). Wet-Etched Microchamber Array Digital PCR Chip for SARS-CoV-2 Virus and Ultra-Early Stage Lung Cancer Quantitative Detection. ACS Omega.

[44] Agha A., Waheed W., Alamoodi N., Mathew B., Alnaimat F., Abu-Nada E., Abderrahmane A., Alazzam A. (2022). A Review of Cyclic Olefin Copolymer Applications in Microfluidics and Microdevices. Macromol. Mater. Eng..

[45] Roper M.G., Easley C.J., Landers J.P. (2005). Advances in Polymerase Chain Reaction on Microfluidic Chips. Anal. Chem..

[46] Arya M., Shergill I.S., Williamson M., Gommersall L., Arya N., Patel H.R.H. (2005). Basic principles of real-time quantitative PCR. Expert Rev. Mol. Diagn..

[47] Kulkarni M.B., Yashas, Vyas R. (2024). A role of integrated microheaters in a microfluidics based point-of-care-testing and beyond for healthcare applications. Appl. Mater. Today.

[48] Moschou D., Vourdas N., Kokkoris G., Papadakis G., Parthenios J., Chatzandroulis S., Tserepi A. (2014). All-plastic, low-power, disposable, continuous-flow PCR chip with integrated microheaters for rapid DNA amplification. Sens. Actuators B Chem..

[49] Beer N.R., Hindson B.J., Wheeler E.K., Hall S.B., Rose K.A., Kennedy I.M., Colston B.W. (2007). On-Chip, Real-Time, Single-Copy Polymerase Chain Reaction in Picoliter Droplets. Anal. Chem..

[50] Zhu H., Fohlerová Z., Pekárek J., Basova E., Neužil P. (2020). Recent advances in lab-on-a-chip technologies for viral diagnosis. Biosens. Bioelectron..

[51] Ranjit Prakash A., Adamia S., Sieben V., Pilarski P., Pilarski L.M., Backhouse C.J. (2006). Small volume PCR in PDMS biochips with integrated fluid control and vapour barrier. Sens. Actuators B Chem..

[52] Matsubara Y., Kerman K., Kobayashi M., Yamamura S., Morita Y., Tamiya E. (2005). Microchamber array based DNA quantification and specific sequence detection from a single copy via PCR in nanoliter volumes. Biosens. Bioelectron..

[53] Yan Z., Jin M., Li Z., Zhou G., Shui L. (2019). Droplet-Based Microfluidic Thermal Management Methods for High Performance Electronic Devices. Micromachines.

[54] Farrar J.S., Wittwer C.T. (2015). Extreme PCR: Efficient and Specific DNA Amplification in 15–60 Seconds. Clin. Chem..

[55] Zhang C., Xing D. (2010). Single-Molecule DNA Amplification and Analysis Using Microfluidics. Chem. Rev..

[56] Afshari Babazad M., Foroozandeh A., Abdouss M., SalarAmoli H., Babazad R.A., Hasanzadeh M. (2024). Recent progress and challenges in biosensing of carcinoembryonic antigen. TrAC Trends Anal. Chem..

[57] de Paz H.D., Brotons P., Muñoz-Almagro C. (2014). Molecular isothermal techniques for combating infectious diseases: Towards low-cost point-of-care diagnostics. Expert Rev. Mol. Diagn..

[58] Shen K., Chen X., Guo M., Cheng J. (2005). A microchip-based PCR device using flexible printed circuit technology. Sens. Actuators B Chem..

[59] Chen S., Sun Y., Fan F., Chen S., Zhang Y., Zhang Y., Meng X., Lin J.-M. (2022). Present status of microfluidic PCR chip in nucleic acid detection and future perspective. TrAC Trends Anal. Chem..

[60] Quan P.-L., Sauzade M., Brouzes E. (2018). dPCR: A Technology Review. Sensors.

[61] Gou T., Hu J., Wu W., Ding X., Zhou S., Fang W., Mu Y. (2018). Smartphone-based mobile digital PCR device for DNA quantitative analysis with high accuracy. Biosens. Bioelectron..

[62] Perkins G., Lu H., Garlan F., Taly V., Makowski G.S. (2017). Chapter Three–Droplet-Based Digital PCR: Application in Cancer Research. Advances in Clinical Chemistry.

[63] Lee S.H., Park S.-m., Kim B.N., Kwon O.S., Rho W.-Y., Jun B.-H. (2019). Emerging ultrafast nucleic acid amplification technologies for next-generation molecular diagnostics. Biosens. Bioelectron..

[64] Wang C., Liu M., Wang Z., Li S., Deng Y., He N. (2021). Point-of-care diagnostics for infectious diseases: From methods to devices. Nano Today.

[65] Nasseri B., Soleimani N., Rabiee N., Kalbasi A., Karimi M., Hamblin M.R. (2018). Point-of-care microfluidic devices for pathogen detection. Biosens. Bioelectron..

[66] Shen F., Du W., Davydova E.K., Karymov M.A., Pandey J., Ismagilov R.F. (2010). Nanoliter Multiplex PCR Arrays on a SlipChip. Anal. Chem..

[67] Zhu Y., Zhang Y.-X., Liu W.-W., Ma Y., Fang Q., Yao B. (2015). Printing 2-Dimentional Droplet Array for Single-Cell Reverse Transcription Quantitative PCR Assay with a Microfluidic Robot. Sci. Rep..

[68] Shao H., Chung J., Lee K., Balaj L., Min C., Carter B.S., Hochberg F.H., Breakefield X.O., Lee H., Weissleder R. (2015). Chip-based analysis of exosomal mRNA mediating drug resistance in glioblastoma. Nat. Commun..

[69] Asiello P.J., Baeumner A.J. (2011). Miniaturized isothermal nucleic acid amplification, a review. Lab Chip.

[70] Zanoli L.M., Spoto G. (2013). Isothermal Amplification Methods for the Detection of Nucleic Acids in Microfluidic Devices. Biosensors.

[71] Zhang Z., Zhao S., Hu F., Yang G., Li J., Tian H., Peng N. (2020). An LED-Driven AuNPs-PDMS Microfluidic Chip and Integrated Device for the Detection of Digital Loop-Mediated Isothermal DNA Amplification. Micromachines.

[72] Yang X., Kui L., Tang M., Li D., Wei K., Chen W., Miao J., Dong Y. (2020). High-Throughput Transcriptome Profiling in Drug and Biomarker Discovery. Front. Genet..

[73] Nero T.L., Morton C.J., Holien J.K., Wielens J., Parker M.W. (2014). Oncogenic protein interfaces: Small molecules, big challenges. Nat. Rev. Cancer.

[74] Verma M., Manne U. (2006). Genetic and epigenetic biomarkers in cancer diagnosis and identifying high risk populations. Crit. Rev. Oncol./Hematol..

[75] Islam M.S., Gopalan V., Lam A.K., Shiddiky M.J.A. (2023). Current advances in detecting genetic and epigenetic biomarkers of colorectal cancer. Biosens. Bioelectron..

[76] Sohrabi H., Bolandi N., Hemmati A., Eyvazi S., Ghasemzadeh S., Baradaran B., Oroojalian F., Reza Majidi M., de la Guardia M., Mokhtarzadeh A. (2022). State-of-the-art cancer biomarker detection by portable (Bio) sensing technology: A critical review. Microchem. J..

[77] Khan N.I., Song E. (2020). Lab-on-a-Chip Systems for Aptamer-Based Biosensing. Micromachines.

[78] Chenarani N., Emamjomeh A., Allahverdi A., Mirmostafa S., Afsharinia M.H., Zahiri J. (2021). Bioinformatic tools for DNA methylation and histone modification: A survey. Genomics.

[79] Goodwin S., McPherson J.D., McCombie W.R. (2016). Coming of age: Ten years of next-generation sequencing technologies. Nat. Rev. Genet..

[80] Li Y., Lee H.J., Corn R.M. (2007). Detection of Protein Biomarkers Using RNA Aptamer Microarrays and Enzymatically Amplified Surface Plasmon Resonance Imaging. Anal. Chem..

[81] Sánchez-Peña M.L., Isaza C.E., Pérez-Morales J., Rodríguez-Padilla C., Castro J.M., Cabrera-Ríos M. (2013). Identification of potential biomarkers from microarray experiments using multiple criteria optimization. Cancer Med..

[82] Pös O., Radvanszky J., Buglyó G., Pös Z., Rusnakova D., Nagy B., Szemes T. (2021). DNA copy number variation: Main characteristics, evolutionary significance, and pathological aspects. Biomed. J..

[83] Reis A.H.O., Vargas F.R., Lemos B. (2016). Biomarkers of genome instability and cancer epigenetics. Tumor Biol..

[84] Pritchard C.C., Salipante S.J., Koehler K., Smith C., Scroggins S., Wood B., Wu D., Lee M.K., Dintzis S., Adey A. (2014). Validation and Implementation of Targeted Capture and Sequencing for the Detection of Actionable Mutation, Copy Number Variation, and Gene Rearrangement in Clinical Cancer Specimens. J. Mol. Diagn..

[85] Kaushik A.M., Hsieh K., Wang T.-H. (2018). Droplet microfluidics for high-sensitivity and high-throughput detection and screening of disease biomarkers. WIREs Nanomed. Nanobiotechnol..

[86] Lundberg M., Thorsen S.B., Assarsson E., Villablanca A., Tran B., Gee N., Knowles M., Nielsen B.S., González Couto E., Martin R. (2011). Multiplexed Homogeneous Proximity Ligation Assays for High-throughput Protein Biomarker Research in Serological Material *. Mol. Cell. Proteom..

[87] Lianidou E. (2021). Detection and relevance of epigenetic markers on ctDNA: Recent advances and future outlook. Mol. Oncol..

[88] Morselli M., Farrell C., Rubbi L., Fehling H.L., Henkhaus R., Pellegrini M. (2021). Targeted bisulfite sequencing for biomarker discovery. Methods.

[89] Reinders J., Paszkowski J. (2010). Bisulfite Methylation Profiling of Large Genomes. Epigenomics.

[90] Carrigan P., Krahn T., Nielsch U., Fuhrmann U., Jaroch S. (2016). Impact of Biomarkers on Personalized Medicine. New Approaches to Drug Discovery.

[91] Baghel R., Maan K., Haritwal T., Rana P., Agrawala P.K., Rana P. (2021). Chapter 2–Integration of epigenomics and metabolomics: From biomarkers discovery to personalized medicine. Epigenetics and Metabolomics.

[92] Uffelmann E., Huang Q.Q., Munung N.S., de Vries J., Okada Y., Martin A.R., Martin H.C., Lappalainen T., Posthuma D. (2021). Genome-wide association studies. Nat. Rev. Methods Primers.

[93] Angelakopoulou A., Shah T., Sofat R., Shah S., Berry D.J., Cooper J., Palmen J., Tzoulaki I., Wong A., Jefferis B.J. (2012). Comparative analysis of genome-wide association studies signals for lipids, diabetes, and coronary heart disease: Cardiovascular Biomarker Genetics Collaboration. Eur. Heart J..

[94] Deming Y., Xia J., Cai Y., Lord J., Del-Aguila J.L., Fernandez M.V., Carrell D., Black K., Budde J., Ma S. (2016). Genetic studies of plasma analytes identify novel potential biomarkers for several complex traits. Sci. Rep..

[95] Broza Y.Y., Zhou X., Yuan M., Qu D., Zheng Y., Vishinkin R., Khatib M., Wu W., Haick H. (2019). Disease Detection with Molecular Biomarkers: From Chemistry of Body Fluids to Nature-Inspired Chemical Sensors. Chem. Rev..

[96] Dhama K., Latheef S.K., Dadar M., Samad H.A., Munjal A., Khandia R., Karthik K., Tiwari R., Yatoo M.I., Bhatt P. (2019). Biomarkers in stress related diseases/disorders: Diagnostic, prognostic, and therapeutic values. Front. Mol. Biosci..

[97] La Thangue N.B., Kerr D.J. (2011). Predictive biomarkers: A paradigm shift towards personalized cancer medicine. Nat. Rev. Clin. Oncol..

[98] Pashayan N., Antoniou A.C., Ivanus U., Esserman L.J., Easton D.F., French D., Sroczynski G., Hall P., Cuzick J., Evans D.G. (2020). Personalized early detection and prevention of breast cancer: ENVISION consensus statement. Nat. Rev. Clin. Oncol..

[99] Passaro A., Al Bakir M., Hamilton E.G., Diehn M., André F., Roy-Chowdhuri S., Mountzios G., Wistuba I.I., Swanton C., Peters S. (2024). Cancer biomarkers: Emerging trends and clinical implications for personalized treatment. Cell.

[100] Li L., Zhang L., Montgomery K.C., Jiang L., Lyon C.J., Hu T.Y. (2023). Advanced technologies for molecular diagnosis of cancer: State of pre-clinical tumor-derived exosome liquid biopsies. Mater. Today Bio.

[101] Sarhadi V.K., Armengol G. (2022). Molecular Biomarkers in Cancer. Biomolecules.

[102] Nicolini A., Ferrari P., Duffy M.J. (2018). Prognostic and predictive biomarkers in breast cancer: Past, present and future. Semin. Cancer Biol..

[103] Markandan K., Tiong Y.W., Sankaran R., Subramanian S., Markandan U.D., Chaudhary V., Numan A., Khalid M., Walvekar R. (2024). Emergence of infectious diseases and role of advanced nanomaterials in point-of-care diagnostics: A review. Biotechnol. Genet. Eng. Rev..

[104] Rosenheim J., Gupta R.K., Thakker C., Mann T., Bell L.C.K., Broderick C.M., Madon K., Papargyris L., Dayananda P., Kwok A.J. (2024). SARS-CoV-2 human challenge reveals biomarkers that discriminate early and late phases of respiratory viral infections. Nat. Commun..

[105] Hong D., Kim K., Jo E.-J., Kim M.-G. (2021). Electrochemiluminescence-Incorporated Lateral Flow Immunosensors Using Ru(bpy)32+-Labeled Gold Nanoparticles for the Full-Range Detection of Physiological C-Reactive Protein Levels. Anal. Chem..

[106] Svegliati-Baroni G., Pierantonelli I., Torquato P., Marinelli R., Ferreri C., Chatgilialoglu C., Bartolini D., Galli F. (2019). Lipidomic biomarkers and mechanisms of lipotoxicity in non-alcoholic fatty liver disease. Free Radic. Biol. Med..

[107] Liu M., Wen Y. (2024). Point-of-care testing for early-stage liver cancer diagnosis and personalized medicine: Biomarkers, current technologies and perspectives. Heliyon.

[108] Stuart T., Satija R. (2019). Integrative single-cell analysis. Nat. Rev. Genet..

[109] Schmid A., Kortmann H., Dittrich P.S., Blank L.M. (2010). Chemical and biological single cell analysis. Curr. Opin. Biotechnol..

[110] Yin H., Marshall D. (2012). Microfluidics for single cell analysis. Curr. Opin. Biotechnol..

[111] Li Y., Ma L., Wu D., Chen G. (2021). Advances in bulk and single-cell multi-omics approaches for systems biology and precision medicine. Brief. Bioinform..

[112] Heath J.R., Ribas A., Mischel P.S. (2016). Single-cell analysis tools for drug discovery and development. Nat. Rev. Drug Discov..

[113] Gu X., Wei S., Lv X. (2024). Circulating tumor cells: From new biological insights to clinical practice. Signal Transduct. Target. Ther..

[114] Hu Y., Shen F., Yang X., Han T., Long Z., Wen J., Huang J., Shen J., Guo Q. (2023). Single-cell sequencing technology applied to epigenetics for the study of tumor heterogeneity. Clin. Epigenetics.

[115] Zhang L., Parvin R., Chen M., Hu D., Fan Q., Ye F. (2023). High-throughput microfluidic droplets in biomolecular analytical system: A review. Biosens. Bioelectron..

[116] Salomon R., Kaczorowski D., Valdes-Mora F., Nordon R.E., Neild A., Farbehi N., Bartonicek N., Gallego-Ortega D. (2019). Droplet-based single cell RNAseq tools: A practical guide. Lab Chip.

[117] Noé A., Cargill T.N., Nielsen C.M., Russell A.J.C., Barnes E. (2020). The Application of Single-Cell RNA Sequencing in Vaccinology. J. Immunol. Res..

[118] Nath A., Bild A.H. (2021). Leveraging Single-Cell Approaches in Cancer Precision Medicine. Trends Cancer.

[119] Chau C.H., Rixe O., McLeod H., Figg W.D. (2008). Validation of Analytic Methods for Biomarkers Used in Drug Development. Clin. Cancer Res..

[120] Kraus V.B. (2018). Biomarkers as drug development tools: Discovery, validation, qualification and use. Nat. Rev. Rheumatol..

[121] Nakayasu E.S., Gritsenko M., Piehowski P.D., Gao Y., Orton D.J., Schepmoes A.A., Fillmore T.L., Frohnert B.I., Rewers M., Krischer J.P. (2021). Tutorial: Best practices and considerations for mass-spectrometry-based protein biomarker discovery and validation. Nat. Protoc..

[122] Klyucherev T.O., Olszewski P., Shalimova A.A., Chubarev V.N., Tarasov V.V., Attwood M.M., Syvänen S., Schiöth H.B. (2022). Advances in the development of new biomarkers for Alzheimer’s disease. Transl. Neurodegener..

[123] Fernández-Metzler C., Ackermann B., Garofolo F., Arnold M.E., DeSilva B., Gu H., Laterza O., Mao Y., Rose M., Vazvaei-Smith F. (2022). Biomarker Assay Validation by Mass Spectrometry. AAPS J..

[124] Vo D.-K., Nguyen T.-T.-L., Maeng H.-J. (2022). Effects of 1α,25-dihydroxyvitamin D3 on the pharmacokinetics and biodistribution of ergothioneine, an endogenous organic cation/carnitine transporter 1 substrate, in rats. J. Pharm. Investig..

[125] Masucci G.V., Cesano A., Hawtin R., Janetzki S., Zhang J., Kirsch I., Dobbin K.K., Alvarez J., Robbins P.B., Selvan S.R. (2016). Validation of biomarkers to predict response to immunotherapy in cancer: Volume I—Pre-analytical and analytical validation. J. ImmunoTherapy Cancer.

[126] Maïno N., Hauling T., Cappi G., Madaboosi N., Dupouy D.G., Nilsson M. (2019). A microfluidic platform towards automated multiplexed in situ sequencing. Sci. Rep..

[127] Godfrey A., Vandendriessche B., Bakker J.P., Fitzer-Attas C., Gujar N., Hobbs M., Liu Q., Northcott C.A., Parks V., Wood W.A. (2021). Fit-for-Purpose Biometric Monitoring Technologies: Leveraging the Laboratory Biomarker Experience. Clin. Transl. Sci..

[128] Perlis R.H. (2011). Translating biomarkers to clinical practice. Mol. Psychiatry.

[129] Chakraborty S. (2024). Democratizing nucleic acid-based molecular diagnostic tests for infectious diseases at resource-limited settings–From point of care to extreme point of care. Sens. Diagn..

[130] Kulasinghe A., Wu H., Punyadeera C., Warkiani M.E. (2018). The Use of Microfluidic Technology for Cancer Applications and Liquid Biopsy. Micromachines.

[131] Mohammadniaei M., Nguyen H.V., Tieu M.V., Lee M.-H. (2019). 2D Materials in Development of Electrochemical Point-of-Care Cancer Screening Devices. Micromachines.

[132] Strom C.M., Rivera S., Elzinga C., Angeloni T., Rosenthal S.H., Goos-Root D., Siaw M., Platt J., Braastadt C., Cheng L. (2015). Development and validation of a next-generation sequencing assay for BRCA1 and BRCA2 variants for the clinical laboratory. PLoS ONE.

[133] Greathouse K.L., White J.R., Vargas A.J., Bliskovsky V.V., Beck J.A., von Muhlinen N., Polley E.C., Bowman E.D., Khan M.A., Robles A.I. (2018). Interaction between the microbiome and TP53 in human lung cancer. Genome Biol..

[134] Brychta N., Krahn T., von Ahsen O. (2016). Detection of KRAS Mutations in Circulating Tumor DNA by Digital PCR in Early Stages of Pancreatic Cancer. Clin. Chem..

[135] Najjar D., Rainbow J., Sharma Timilsina S., Jolly P., de Puig H., Yafia M., Durr N., Sallum H., Alter G., Li J.Z. (2022). A lab-on-a-chip for the concurrent electrochemical detection of SARS-CoV-2 RNA and anti-SARS-CoV-2 antibodies in saliva and plasma. Nat. Biomed. Eng..

[136] Nouri R., Jiang Y., Politza A.J., Liu T., Greene W.H., Zhu Y., Nunez J.J., Lian X., Guan W. (2023). STAMP-Based Digital CRISPR-Cas13a for Amplification-Free Quantification of HIV-1 Plasma Viral Loads. ACS Nano.

[137] Fang X., Zheng Y., Duan Y., Liu Y., Zhong W. (2019). Recent Advances in Design of Fluorescence-Based Assays for High-Throughput Screening. Anal. Chem..

[138] Li Z., Zhao J., Wu X., Zhu C., Liu Y., Wang A., Deng G., Zhu L. (2019). A rapid microfluidic platform with real-time fluorescence detection system for molecular diagnosis. Biotechnol. Biotechnol. Equip..

[139] Wang R.C., Wang Z. (2023). Precision Medicine: Disease Subtyping and Tailored Treatment. Cancers.

[140] Chen F., Wang J., Ma J., Song L., Yan H., Wang F., Yang Z., Li F. (2024). Novel DNA Biosensing Platform for Detecting HIV Integrase for Highly Sensitive and Quantitative HIV Detection, Diagnosis, and Therapeutic Monitoring. ACS Omega.

[141] Sefrioui D., Sarafan-Vasseur N., Beaussire L., Baretti M., Gangloff A., Blanchard F., Clatot F., Sabourin J.-C., Sesboüé R., Frebourg T. (2015). Clinical value of chip-based digital-PCR platform for the detection of circulating DNA in metastatic colorectal cancer. Dig. Liver Dis..

[142] Lee J.-m., Han J.J., Altwerger G., Kohn E.C. (2011). Proteomics and biomarkers in clinical trials for drug development. J. Proteom..

[143] Hopkins A.L., Groom C.R. (2002). The druggable genome. Nat. Rev. Drug Discov..

[144] Das S., Dey M.K., Devireddy R., Gartia M.R. (2024). Biomarkers in Cancer Detection, Diagnosis, and Prognosis. Sensors.

[145] Haghayegh F., Norouziazad A., Haghani E., Feygin A.A., Rahimi R.H., Ghavamabadi H.A., Sadighbayan D., Madhoun F., Papagelis M., Felfeli T. (2024). Revolutionary Point-of-Care Wearable Diagnostics for Early Disease Detection and Biomarker Discovery through Intelligent Technologies. Adv. Sci..

[146] Levantini E., Maroni G., Del Re M., Tenen D.G. (2022). EGFR signaling pathway as therapeutic target in human cancers. Semin. Cancer Biol..

[147] Rascio F., Spadaccino F., Rocchetti M.T., Castellano G., Stallone G., Netti G.S., Ranieri E. (2021). The Pathogenic Role of PI3K/AKT Pathway in Cancer Onset and Drug Resistance: An Updated Review. Cancers.

[148] Li M., Chi X., Wang Y., Setrerrahmane S., Xie W., Xu H. (2022). Trends in insulin resistance: Insights into mechanisms and therapeutic strategy. Signal Transduct. Target. Ther..

[149] Wang D.-R., Wu X.-L., Sun Y.-L. (2022). Therapeutic targets and biomarkers of tumor immunotherapy: Response versus non-response. Signal Transduct. Target. Ther..

[150] Nakamura Y., Kawazoe A., Lordick F., Janjigian Y.Y., Shitara K. (2021). Biomarker-targeted therapies for advanced-stage gastric and gastro-oesophageal junction cancers: An emerging paradigm. Nat. Rev. Clin. Oncol..

[151] Monette A., Aguilar-Mahecha A., Altinmakas E., Angelos M.G., Assad N., Batist G., Bommareddy P.K., Bonilla D.L., Borchers C.H., Church S.E. (2024). The Society for Immunotherapy of Cancer Perspective on Tissue-Based Technologies for Immuno-Oncology Biomarker Discovery and Application. Clin. Cancer Res..

[152] Wang X., Collet L., Rediti M., Debien V., De Caluwé A., Venet D., Romano E., Rothé F., Sotiriou C., Buisseret L. (2023). Predictive Biomarkers for Response to Immunotherapy in Triple Negative Breast Cancer: Promises and Challenges. J. Clin. Med..

[153] Holmes M.V., Richardson T.G., Ference B.A., Davies N.M., Davey Smith G. (2021). Integrating genomics with biomarkers and therapeutic targets to invigorate cardiovascular drug development. Nat. Rev. Cardiol..

[154] Qiu S., Cai Y., Yao H., Lin C., Xie Y., Tang S., Zhang A. (2023). Small molecule metabolites: Discovery of biomarkers and therapeutic targets. Signal Transduct. Target. Ther..

[155] Emmerich C.H., Gamboa L.M., Hofmann M.C.J., Bonin-Andresen M., Arbach O., Schendel P., Gerlach B., Hempel K., Bespalov A., Dirnagl U. (2021). Improving target assessment in biomedical research: The GOT-IT recommendations. Nat. Rev. Drug Discov..

[156] Middleton G., Robbins H., Andre F., Swanton C. (2022). A state-of-the-art review of stratified medicine in cancer: Towards a future precision medicine strategy in cancer. Ann. Oncol..

[157] Lawler M., Keeling P., Kholmanskikh O., Minnaard W., Moehlig-Zuttermeister H., Normanno N., Philip R., Popp C., Salgado R., Santiago-Walker A.E. (2024). Empowering effective biomarker-driven precision oncology: A call to action. Eur. J. Cancer.

[158] Zhou Z., Lin T., Chen S., Zhang G., Xu Y., Zou H., Zhou A., Zhang Y., Weng S., Han X. (2024). Omics-based molecular classifications empowering in precision oncology. Cell. Oncol..

[159] Swain S.M., Shastry M., Hamilton E. (2023). Targeting HER2-positive breast cancer: Advances and future directions. Nat. Rev. Drug Discov..

[160] Aldea M., Andre F., Marabelle A., Dogan S., Barlesi F., Soria J.-C. (2021). Overcoming resistance to tumor-targeted and immune-targeted therapies. Cancer Discov..

[161] Williams D.M., Jones H., Stephens J.W. (2022). Personalized Type 2 Diabetes Management: An Update on Recent Advances and Recommendations. Diabetes Metab. Syndr. Obes..

[162] Ponce D.M., Alousi A.M., Nakamura R., Slingerland J., Calafiore M., Sandhu K.S., Barker J.N., Devlin S., Shia J., Giralt S. (2023). A phase 2 study of interleukin-22 and systemic corticosteroids as initial treatment for acute GVHD of the lower GI tract. Blood.

[163] Zanella E.R., Grassi E., Trusolino L. (2022). Towards precision oncology with patient-derived xenografts. Nat. Rev. Clin. Oncol..

[164] Di Nicolantonio F., Vitiello P.P., Marsoni S., Siena S., Tabernero J., Trusolino L., Bernards R., Bardelli A. (2021). Precision oncology in metastatic colorectal cancer—From biology to medicine. Nat. Rev. Clin. Oncol..

[165] Muthamilselvan S., Ramasami Sundhar Baabu P., Palaniappan A. (2023). Microfluidics for Profiling miRNA Biomarker Panels in AI-Assisted Cancer Diagnosis and Prognosis. Technol. Cancer Res. Treat..

[166] Al-Thani A.N., Jan A.G., Abbas M., Geetha M., Sadasivuni K.K. (2024). Nanoparticles in cancer theragnostic and drug delivery: A comprehensive review. Life Sci..

[167] Normanno N., Apostolidis K., de Lorenzo F., Beer P.A., Henderson R., Sullivan R., Biankin A.V., Horgan D., Lawler M. (2022). Cancer Biomarkers in the era of precision oncology: Addressing the needs of patients and health systems. Semin. Cancer Biol..

[168] Sanz-Garcia E., Zhao E., Bratman S.V., Siu L.L. (2022). Monitoring and adapting cancer treatment using circulating tumor DNA kinetics: Current research, opportunities, and challenges. Sci. Adv..

[169] Pascual J., Attard G., Bidard F.C., Curigliano G., De Mattos-Arruda L., Diehn M., Italiano A., Lindberg J., Merker J.D., Montagut C. (2022). ESMO recommendations on the use of circulating tumour DNA assays for patients with cancer: A report from the ESMO Precision Medicine Working Group. Ann. Oncol..

[170] Parkins Michael D., Lee Bonita E., Acosta N., Bautista M., Hubert Casey R.J., Hrudey Steve E., Frankowski K., Pang X.-L. (2023). Wastewater-based surveillance as a tool for public health action: SARS-CoV-2 and beyond. Clin. Microbiol. Rev..

[171] Wang S., Li H., Kou Z., Ren F., Jin Y., Yang L., Dong X., Yang M., Zhao J., Liu H. (2021). Highly sensitive and specific detection of hepatitis B virus DNA and drug resistance mutations utilizing the PCR-based CRISPR-Cas13a system. Clin. Microbiol. Infect..

[172] Aulin L.B.S., de Lange D.W., Saleh M.A.A., van der Graaf P.H., Völler S., van Hasselt J.G.C. (2021). Biomarker-Guided Individualization of Antibiotic Therapy. Clin. Pharmacol. Ther..

[173] Vo D.-K., Nguyen T.-T.-L., Maeng H.-J. (2024). Impact of 1α,25-dihydroxyvitamin D3 on biodistribution and pharmacokinetics of L-carnitine and creatinine, organic cation/carnitine transporter 2 and organic cation transporter 2 biomarkers. J. Pharm. Investig..

[174] Xiao H., Wang X., Li S., Liu Y., Cui Y., Deng X. (2021). Advances in Biomarkers for Detecting Early Cancer Treatment-Related Cardiac Dysfunction. Front. Cardiovasc. Med..

[175] Sharma R., Singh D., Gaur P., Joshi D. (2021). Intelligent automated drug administration and therapy: Future of healthcare. Drug Deliv. Transl. Res..

[176] Valla V., Alzabin S., Koukoura A., Lewis A., Nielsen A.A., Vassiliadis E. (2021). Companion Diagnostics: State of the Art and New Regulations. Biomark. Insights.

[177] Orellana García L.P., Ehmann F., Hines P.A., Ritzhaupt A., Brand A. (2021). Biomarker and Companion Diagnostics—A Review of Medicinal Products Approved by the European Medicines Agency. Front. Med..

[178] Bakker E., Starokozhko V., Kraaijvanger J.W.M., Heerspink H.J.L., Mol P.G.M. (2023). Precision medicine in regulatory decision making: Biomarkers used for patient selection in European Public Assessment Reports from 2018 to 2020. Clin. Transl. Sci..

[179] Arafah A., Khatoon S., Rasool I., Khan A., Rather M.A., Abujabal K.A., Faqih Y.A., Rashid H., Rashid S.M., Bilal Ahmad S. (2023). The Future of Precision Medicine in the Cure of Alzheimer’s Disease. Biomedicines.

[180] Pal M., Muinao T., Boruah H.P.D., Mahindroo N. (2022). Current advances in prognostic and diagnostic biomarkers for solid cancers: Detection techniques and future challenges. Biomed. Pharmacother..

[181] Marques L., Costa B., Pereira M., Silva A., Santos J., Saldanha L., Silva I., Magalhães P., Schmidt S., Vale N. (2024). Advancing Precision Medicine: A Review of Innovative In Silico Approaches for Drug Development, Clinical Pharmacology and Personalized Healthcare. Pharmaceutics.

[182] Bartel P., Granger J. (2024). Introduction to Companion Diagnostics for Gene Therapy. Drug Development for Gene Therapy.

[183] Acharya D., Mukhopadhyay A. (2024). A comprehensive review of machine learning techniques for multi-omics data integration: Challenges and applications in precision oncology. Brief. Funct. Genom..

[184] Tolani P., Gupta S., Yadav K., Aggarwal S., Yadav A.K., Donev R., Karabencheva-Christova T. (2021). Chapter Four–Big data, integrative omics and network biology. Advances in Protein Chemistry and Structural Biology.

[185] Caudai C., Galizia A., Geraci F., Le Pera L., Morea V., Salerno E., Via A., Colombo T. (2021). AI applications in functional genomics. Comput. Struct. Biotechnol. J..

[186] Bahl A., Ibrahim C., Plate K., Haase A., Dengjel J., Nymark P., Dumit V.I. (2023). PROTEOMAS: A workflow enabling harmonized proteomic meta-analysis and proteomic signature mapping. J. Cheminformatics.

[187] Zhou Z., Zhang R., Zhou A., Lv J., Chen S., Zou H., Zhang G., Lin T., Wang Z., Zhang Y. (2024). Proteomics appending a complementary dimension to precision oncotherapy. Comput. Struct. Biotechnol. J..

[188] Russell A.M., Pack A.P., Bailey S.C., Weldon C.B., Dreyer M.S., Kircher S.M., Wolf M.S. (2024). A local perspective on internal, external, and reflexive biomarker testing processes for lung cancer in an academic medical center. Cancer.

[189] Kaur P., Singh A., Chana I. (2021). Computational Techniques and Tools for Omics Data Analysis: State-of-the-Art, Challenges, and Future Directions. Arch. Comput. Methods Eng..

[190] Kim S., Kang S., Choe J., Moon C., Choi H., Kim J.-Y., Choi J.-W. (2023). A Microfluidic System for Investigating Anticipatory Medication Effects on Dopamine Homeostasis in Dopaminergic Cells. Anal. Chem..

[191] Batis N., Brooks J.M., Payne K., Sharma N., Nankivell P., Mehanna H. (2021). Lack of predictive tools for conventional and targeted cancer therapy: Barriers to biomarker development and clinical translation. Adv. Drug Deliv. Rev..

[192] Moqri M., Herzog C., Poganik J.R., Ying K., Justice J.N., Belsky D.W., Higgins-Chen A.T., Chen B.H., Cohen A.A., Fuellen G. (2024). Validation of biomarkers of aging. Nat. Med..

[193] Guo L., Kong D., Liu J., Zhan L., Luo L., Zheng W., Zheng Q., Chen C., Sun S. (2023). Breast cancer heterogeneity and its implication in personalized precision therapy. Exp. Hematol. Oncol..

[194] Lenz G., Onzi G.R., Lenz L.S., Buss J.H., dos Santos J.A., Begnini K.R. (2022). The Origins of Phenotypic Heterogeneity in Cancer. Cancer Res..

[195] Mani D.R., Krug K., Zhang B., Satpathy S., Clauser K.R., Ding L., Ellis M., Gillette M.A., Carr S.A. (2022). Cancer proteogenomics: Current impact and future prospects. Nat. Rev. Cancer.

[196] Ghosh S., Rajendran R.L., Mahajan A.A., Chowdhury A., Bera A., Guha S., Chakraborty K., Chowdhury R., Paul A., Jha S. (2024). Harnessing exosomes as cancer biomarkers in clinical oncology. Cancer Cell Int..

[197] del Campo M., Zetterberg H., Gandy S., Onyike C.U., Oliveira F., Udeh-Momoh C., Lleó A., Teunissen C.E., Pijnenburg Y. (2022). New developments of biofluid-based biomarkers for routine diagnosis and disease trajectories in frontotemporal dementia. Alzheimer’s Dement..

[198] Nguyen H.T., Peirsman A., Tirpakova Z., Mandal K., Vanlauwe F., Maity S., Kawakita S., Khorsandi D., Herculano R., Umemura C. (2023). Engineered Vasculature for Cancer Research and Regenerative Medicine. Micromachines.

[199] Füzéry A.K., Levin J., Chan M.M., Chan D.W. (2013). Translation of proteomic biomarkers into FDA approved cancer diagnostics: Issues and challenges. Clin. Proteom..

[200] Kang S.L., Woo J.H., Kim N.H., Kwon J.Y., Kim S.M. (2023). Necessity of strengthening the current clinical regulatory for companion diagnostics: An institutional comparison of the FDA, EMA, and MFDS. Mol. Ther. Methods Clin. Dev..

[201] Bakker E., Hendrikse N.M., Ehmann F., van der Meer D.S., Llinares Garcia J., Vetter T., Starokozhko V., Mol P.G.M. (2022). Biomarker Qualification at the European Medicines Agency: A Review of Biomarker Qualification Procedures From 2008 to 2020. Clin. Pharmacol. Ther..

[202] Zwanenburg A., Vallières M., Abdalah M.A., Aerts H.J.W.L., Andrearczyk V., Apte A., Ashrafinia S., Bakas S., Beukinga R.J., Boellaard R. (2020). The image biomarker standardization initiative: Standardized quantitative radiomics for high-throughput image-based phenotyping. Radiology.

[203] Gromova M., Vaggelas A., Dallmann G., Seimetz D. (2020). Biomarkers: Opportunities and challenges for drug development in the current regulatory landscape. Biomark. Insights.

[204] Kingsmore S.F. (2006). Multiplexed protein measurement: Technologies and applications of protein and antibody arrays. Nat. Rev. Drug Discov..

[205] Hays A., Wissel M., Colletti K., Soon R., Azadeh M., Smith J., Doddareddy R., Chalfant M., Adamowicz W., Ramaswamy S.S. (2024). Recommendations for Method Development and Validation of qPCR and dPCR Assays in Support of Cell and Gene Therapy Drug Development. AAPS J..

[206] Tenchov R., Sapra A.K., Sasso J., Ralhan K., Tummala A., Azoulay N., Zhou Q.A. (2024). Biomarkers for Early Cancer Detection: A Landscape View of Recent Advancements, Spotlighting Pancreatic and Liver Cancers. ACS Pharmacol. Transl. Sci..

[207] Agarwal A., Ressler D., Snyder G. (2015). The current and future state of companion diagnostics. Pharmacogenomics Pers. Med..

[208] Kim S.Y.H., Karlawish J., Berkman B.E. (2015). Ethics of genetic and biomarker test disclosures in neurodegenerative disease prevention trials. Neurology.

[209] Erdmann A., Rehmann-Sutter C., Bozzaro C. (2021). Patients’ and professionals’ views related to ethical issues in precision medicine: A mixed research synthesis. BMC Med. Ethics.

[210] Singhal P., Tan A.L.M., Drivas T.G., Johnson K.B., Ritchie M.D., Beaulieu-Jones B.K. (2023). Opportunities and challenges for biomarker discovery using electronic health record data. Trends Mol. Med..

[211] Avilés-Santa M.L., Heintzman J., Lindberg N.M., Guerrero-Preston R., Ramos K., Abraído-Lanza A.L., Bull J., Falcón A., McBurnie M.A., Moy E. (2017). Personalized medicine and Hispanic health: Improving health outcomes and reducing health disparities—A National Heart, Lung, and Blood Institute workshop report. BMC Proc..

[212] Jurjako M., Malatesti L., Brazil I.A. (2019). Some Ethical Considerations About the Use of Biomarkers for the Classification of Adult Antisocial Individuals. Int. J. Forensic Ment. Health.

[213] Walsh C.G., Chaudhry B., Dua P., Goodman K.W., Kaplan B., Kavuluru R., Solomonides A., Subbian V. (2020). Stigma, biomarkers, and algorithmic bias: Recommendations for precision behavioral health with artificial intelligence. JAMIA Open.

[214] Brannan C., Foulkes A.L., Lázaro-Muñoz G. (2019). Preventing discrimination based on psychiatric risk biomarkers. Am. J. Med. Genet. Part B Neuropsychiatr. Genet..

[215] Khera R., Oikonomou E.K., Nadkarni G.N., Morley J.R., Wiens J., Butte A.J., Topol E.J. (2024). Transforming cardiovascular care with artificial intelligence: From discovery to practice: JACC state-of-the-art review. J. Am. Coll. Cardiol..

[216] Wang B., Li Y., Zhou M., Han Y., Zhang M., Gao Z., Liu Z., Chen P., Du W., Zhang X. (2023). Smartphone-based platforms implementing microfluidic detection with image-based artificial intelligence. Nat. Commun..

[217] Gupta R., Srivastava D., Sahu M., Tiwari S., Ambasta R.K., Kumar P. (2021). Artificial intelligence to deep learning: Machine intelligence approach for drug discovery. Mol. Divers..

[218] Shapiro M.R., Tallon E.M., Brown M.E., Posgai A.L., Clements M.A., Brusko T.M. (2024). Leveraging artificial intelligence and machine learning to accelerate discovery of disease-modifying therapies in type 1 diabetes. Diabetologia.

[219] Doherty T., Yao Z., Khleifat A.A.l., Tantiangco H., Tamburin S., Albertyn C., Thakur L., Llewellyn D.J., Oxtoby N.P., Lourida I. (2023). Artificial intelligence for dementia drug discovery and trials optimization. Alzheimer’s Dement..

[220] Prelaj A., Miskovic V., Zanitti M., Trovo F., Genova C., Viscardi G., Rebuzzi S.E., Mazzeo L., Provenzano L., Kosta S. (2024). Artificial intelligence for predictive biomarker discovery in immuno-oncology: A systematic review. Ann. Oncol..

[221] Joshi R.C., Srivastava P., Mishra R., Burget R., Dutta M.K. (2024). Biomarker profiling and integrating heterogeneous models for enhanced multi-grade breast cancer prognostication. Comput. Methods Programs Biomed..

[222] Sahiner B., Pezeshk A., Hadjiiski L.M., Wang X., Drukker K., Cha K.H., Summers R.M., Giger M.L. (2019). Deep learning in medical imaging and radiation therapy. Med. Phys..

[223] Odenkirk M.T., Reif D.M., Baker E.S. (2021). Multiomic Big Data Analysis Challenges: Increasing Confidence in the Interpretation of Artificial Intelligence Assessments. Anal. Chem..

[224] Chakraborty S., Sharma G., Karmakar S., Banerjee S. (2024). Multi-OMICS approaches in cancer biology: New era in cancer therapy. Biochim. Biophys. Acta (BBA) Mol. Basis Dis..

[225] Barrat F.J., Crow M.K., Ivashkiv L.B. (2019). Interferon target-gene expression and epigenomic signatures in health and disease. Nat. Immunol..

[226] Aebersold R., Mann M. (2016). Mass-spectrometric exploration of proteome structure and function. Nature.

[227] Song Y., Xu X., Wang W., Tian T., Zhu Z., Yang C. (2019). Single cell transcriptomics: Moving towards multi-omics. Analyst.

[228] Bargahi N., Ghasemali S., Jahandar-Lashaki S., Nazari A. (2022). Recent advances for cancer detection and treatment by microfluidic technology, review and update. Biol. Proced. Online.

[229] Sanchez-Freire V., Ebert A.D., Kalisky T., Quake S.R., Wu J.C. (2012). Microfluidic single-cell real-time PCR for comparative analysis of gene expression patterns. Nat. Protoc..

[230] Labib M., Kelley S.O. (2020). Single-cell analysis targeting the proteome. Nat. Rev. Chem..

[231] Bennett H.M., Stephenson W., Rose C.M., Darmanis S. (2023). Single-cell proteomics enabled by next-generation sequencing or mass spectrometry. Nat. Methods.

[232] Özyurt C., Uludağ İ., İnce B., Sezgintürk M.K. (2023). Lab-on-a-chip systems for cancer biomarker diagnosis. J. Pharm. Biomed. Anal..

[233] Chan H.N., Tan M.J.A., Wu H. (2017). Point-of-care testing: Applications of 3D printing. Lab Chip.

[234] Prabhakar P., Sen R.K., Dwivedi N., Khan R., Solanki P.R., Srivastava A.K., Dhand C. (2021). 3D-Printed Microfluidics and Potential Biomedical Applications. Front. Nanotechnol..

[235] Mao M., He J., Li X., Zhang B., Lei Q., Liu Y., Li D. (2017). The Emerging Frontiers and Applications of High-Resolution 3D Printing. Micromachines.

[236] Chavez-Pineda O.G., Rodriguez-Moncayo R., Cedillo-Alcantar D.F., Guevara-Pantoja P.E., Amador-Hernandez J.U., Garcia-Cordero J.L. (2022). Microfluidic systems for the analysis of blood-derived molecular biomarkers. Electrophoresis.

[237] Sharma S., Zapatero-Rodríguez J., Estrela P., Kennedy R. (2015). Point-of-Care Diagnostics in Low Resource Settings: Present Status and Future Role of Microfluidics. Biosensors.

[238] Dkhar D.S., Kumari R., Malode S.J., Shetti N.P., Chandra P. (2023). Integrated lab-on-a-chip devices: Fabrication methodologies, transduction system for sensing purposes. J. Pharm. Biomed. Anal..

[239] Ríos Á., Zougagh M., Avila M. (2012). Miniaturization through lab-on-a-chip: Utopia or reality for routine laboratories? A review. Anal. Chim. Acta.

[240] Clack K., Soda N., Kasetsirikul S., Mahmudunnabi R.G., Nguyen N.-T., Shiddiky M.J.A. (2023). Toward Personalized Nanomedicine: The Critical Evaluation of Micro and Nanodevices for Cancer Biomarker Analysis in Liquid Biopsy. Small.

[241] Jain S., Nehra M., Kumar R., Dilbaghi N., Hu T., Kumar S., Kaushik A., Li C.-z. (2021). Internet of medical things (IoMT)-integrated biosensors for point-of-care testing of infectious diseases. Biosens. Bioelectron..

[242] Chu H., Liu C., Liu J., Yang J., Li Y., Zhang X. (2021). Recent advances and challenges of biosensing in point-of-care molecular diagnosis. Sens. Actuators B Chem..

[243] Gardy J.L., Loman N.J. (2018). Towards a genomics-informed, real-time, global pathogen surveillance system. Nat. Rev. Genet..

[244] Ielapi N., Andreucci M., Licastro N., Faga T., Grande R., Buffone G., Mellace S., Sapienza P., Serra R. (2020). Precision Medicine and Precision Nursing: The Era of Biomarkers and Precision Health. Int. J. Gen. Med..

[245] Kelloff G.J., Sigman C.C. (2012). Cancer biomarkers: Selecting the right drug for the right patient. Nat. Rev. Drug Discov..

[246] Herder C., Roden M. (2022). A novel diabetes typology: Towards precision diabetology from pathogenesis to treatment. Diabetologia.

[247] Walzl G., McNerney R., du Plessis N., Bates M., McHugh T.D., Chegou N.N., Zumla A. (2018). Tuberculosis: Advances and challenges in development of new diagnostics and biomarkers. Lancet Infect. Dis..

[248] Zarei M. (2017). Advances in point-of-care technologies for molecular diagnostics. Biosens. Bioelectron..

